# Expansion of Imaginal Disc Growth Factor Gene Family in Diptera Reflects the Evolution of Novel Functions

**DOI:** 10.3390/insects10100365

**Published:** 2019-10-20

**Authors:** Martina Zurovcova, Vladimir Benes, Michal Zurovec, Lucie Kucerova

**Affiliations:** 1 Biology Centre, Czech Academy of Sciences, Institute of Entomology, Branisovska 31, 370 05 Ceske Budejovice, Czech Republic; m.zurovcova@seznam.cz (M.Z.); zurovec@entu.cas.cz (M.Z.); 2 European Molecular Biology Laboratory (EMBL), Core Facilities and Services, Meyerhofstraße 1, 69117 Heidelberg, Germany; benes@embl.de; 3 Faculty of Science, University of South Bohemia, Branisovska 1760, 370 05 Ceske Budejovice, Czech Republic

**Keywords:** *Drosophila*, chitinase, phylogeny, positive selection, chitinase-like protein, IDGF

## Abstract

Imaginal disc growth factors (IDGFs) are a small protein family found in insects. They are related to chitinases and implicated in multiple functions, including cell growth stimulation, antimicrobial activity, insect hemolymph clotting, and maintenance of the extracellular matrix. A number of new IDGFs have been found in several insect species and their detailed phylogenetic analysis provides a good basis for further functional studies. To achieve this goal, we sequenced *Idgf* cDNAs from several lepidopteran and trichopteran species and supplemented our data with sequences retrieved from public databases. A comparison of *Idgf* genes in different species showed that Diptera typically contain several *Idgf* paralogs with a simple exon-intron structure (2–3 exons), whereas lepidopteran *Idgfs* appear as a single copy per genome and contain a higher number of exons (around 9). Our results show that, while lepidopteran *Idgfs*, having single orthologs, are characterized by low divergence and stronger purifying selection over most of the molecule, the duplicated *Idgf* genes in Diptera, *Idgf1* and *Idgf4,* exhibit signs of positive selection. This characterization of IDGF evolution provides, to our knowledge, the first information on the changes that formed these important molecules.

## 1. Introduction

Imaginal disc growth factors (IDGFs) are a small glycoprotein family of chitinase-related secretory proteins found in a number of insect species. They were originally discovered as mitogenic factors produced by *Drosophila melanogaster* imaginal disc and embryonic cell lines [1,2]. Similar glycoproteins are found in other insects and have been implicated in antimicrobial responses and insect hemolymph clotting, as well as the molting process and extracellular matrix formation [3,4,5,6]. Recent studies on *Aedes aegypti* IDGF orthologs also suggest that these proteins are present in mosquito saliva and may be involved in the modulation of the mammalian host response and enhance mosquito-borne Zika virus pathogenesis in mice [7].

Structurally, IDGFs belong to glycosyl-hydrolase family 18, in which they are classified as Group V of chitinases. IDGFs differ from canonical chitinase enzymes by an extra loop sequence (24 extra amino acid residues) between β-sheet 4 and α-helix 4. In addition, they contain an amino acid substitution of a key glutamic acid residue in the hydrolase active centre; therefore, IDGFs do not possess enzymatic chitinase activity [8]. They may, however, still bind some carbohydrate moieties and function as lectins. The three-dimensional structure of one IDGF family member, IDGF2, has been determined at 1.3 Å resolution by X-ray analysis, revealing the characteristic (βα)_8_ or triose-phosphate isomerase barrel-fold of glycosyl-hydrolase family 18 [8].

IDGFs have been detected in a number of insect species, showing approximately 50% similarity at the amino acid level. IDGFs are mainly produced by the fat body and haemocytes, but are present in all tissues and developmental stages and, in some species, they can be induced by ecdysone or injury [1,9,10]. Our recent study showed that IDGFs are also expressed in the silk glands and secreted into the silk produced by lepidopteran caterpillars [11]. Some insect species, especially those within Diptera, contain multiple IDGF paralogs with at least partially overlapping functions, which may be connected to the evolution of new functions [12]. Deciphering the relationships of IDGF proteins is a critical step toward understanding their function in insects.

To examine the evolutionary history of IDGFs, we constructed a phylogenetic tree based on sequence data derived from our research, supplemented with sequences from public databases. We tested for signatures of natural selection on *Idgf* genes at both the gene and protein product level, using a variety of statistical methods based on the ratio of synonymous to non-synonymous substitutions across dipteran and lepidopteran insect lineages. Our results show that the majority of the *Idgf* coding sequence is under purifying selection, which is connected to stabilization of the conserved (βα)_8_ barrel structure typical for the 18 glycosyl-hydrolase family. However, we also identified signatures of positive selection and accelerated evolutionary rate, specifically in Schizophora (higher Diptera) IDGFs, connected to changes in the polarity of the protein and solvent accessibility of some specific regions of the molecule.

## 2. Materials and Methods

### 2.1. Transcriptome Preparation

Total RNA samples from dissected larval silk glands of *Tineola bisselliella*, *Acanthobrahmaea europaea*, *Hepialus humuli*, *Eumeta pryeri*, *Yponomeuta cagnagella*, *Thaumatopoea pithyocampa*, *Plectrocnemia conspersa*, *Phyllonorycter roboris*, *Lymantria dispar*, *Andraca theae* (Order Lepidoptera), and *Hydropsyche angustipennis* (Order Trichoptera) were extracted using Trizol reagent (Life Technologies, Carlsbad, CA, USA) and used to prepare cDNA libraries for the Illumina sequencing platform, as described previously [13]. In addition, cDNA libraries from four other lepidopteran species, *Malacosoma neustria*, *Lasiocampa quercus*, *Arctia caja*, and *Bena prasinana*, as well as two caddisflies, *Oligotricha striata* and *Rhyacophila obliterate* (Order Trichoptera), were sequenced using Roche/454 sequencing technology, as described earlier [14]. A RiboMinus Eukaryote Kit for RNA-Seq (Ambion, Austin, TX, USA) was used to remove rRNA, after which the poly-A mRNA was enriched with the aid of a Dynabeads Oligo (dT)_25_ mRNA Purification Kit (Thermo Fisher Scientific, Waltham, Ma, USA), and the cDNA library was created with a NEXTflex Rapid RNA-Seq Kit (Bioo Scientific, Austin, TX, USA), as described earlier [13]. The sequencing was performed with a MiSeq (Illumina, San Diego, CA, USA) instrument, producing sequences in the 2 × 150 nt pair-end format. Reads were assembled de novo using Trinity software (the default options and a minimum allowed length of 200 bp) [15] on the Galaxy platform [16]. *Idgf* cDNAs in the transcriptomes of all examined species were detected using a local BLAST search and previously identified *Idgfs* as queries. The sequences were deposited in GenBank (see Table S1). 

### 2.2. Phylogenetic Analysis of IDGFs

The sequences of putative IDGF proteins were used to construct tentative phylogenetic trees. The coding nucleotide sequences were aligned as codons using MUSCLE software [17]. GTR + G + I was chosen as the best model for phylogenetic reconstruction using smart model selection (SMS) [18] according to the lowest Bayesian information criterion (BIC) and Akaike information criterion (AIC) scores. Phylogenetic analysis was performed in MEGA 6 software [19] using the minimum evolution (ME) tree reconstruction method (branch support was verified by bootstrap analysis, 10,000 replicates), followed by analysis in PhyML v.3.0 using the maximum-likelihood (ML) method using aBayes branch support test [20], as well as the Bayesian inference (BI) method implemented in MrBayes v.3.2.6.21 [21]. Phylogenetic trees were finalized with the aid of Figtree v.1.4.3 [22].

### 2.3. Molecular Evolution Analysis

Separate analyses were run for each of the duplicated genes within the Schizophora lineage, with single copy *Idgf* from lower Diptera (*Mayetiola destructor*), *BRP1* from *Anopheles gambiae*, and *Idgf* from the *Ctenocephalides felis* (Order Siphonaptera) as outgroups. For comparison, analyses of the single copy genes from Lepidoptera and Trichoptera were conducted; in this case, we used *Idgf3* from *D. melanogaster*, *BRP1* from *An. gambiae*, and *Idgf* from *C. felis* as outgroups. Selection was also tested within the wider phylogenetic frame, namely through comparison of *Idgfs* from both Trichoptera and Lepidoptera with those of Diptera and Siphonaptera; in this larger data set, only the presumed ancestral *Idgf4* from the Schizophora group was used.

### 2.4. Gene-Level Approach Tests

First, tests for positive selection at specific codons were performed utilizing Selecton software v.2.4 (http://selecton.tau.ac.il) [23]. We applied the codon-based mechanistic-empirical combination (MEC) evolutionary model of Doron-Faigenboim and Pupko [24], which takes into account the differences between diverse amino acid replacement probabilities. Where a significant positive selection was detected, it was verified by comparison of the Akaike information criterion (AIC) score of this model with the AIC score obtained under the M8a null model of Swanson et al. [25], as recommended by the authors.

To search for the possible impact of selection acting along the *Idgf* phylogeny, additional codon models were employed on the Datamonkey web server (http://www.datamonkey.org/) [26], implementing five different methods: (1) mixed effects model of evolution (MEME) [27,28], to test the hypothesis that individual sites have been subjected to episodic positive or diversifying selection; (2) fixed effects likelihood (FEL) [26] and (3) fast unconstrained Bayesian approximation (FUBAR) [27], to test the hypothesis that individual sites have been subjected to pervasive positive or purifying selection; (4) aBSREL (adaptive branch-site random effects likelihood) [29,30], to test each lineage for episodic selection in an exploratory fashion; and (5) RELAX [31], to test if the gene-wide selection pressure has been relaxed or intensified along a focal subset of branches.

A significance level of *p* < 0.1 in both MEME and FEL, and Bayes posterior probability >0.9 in FUBAR, were considered to indicate positively *selected* sites; in aBSREL, *p* ≤ 0.05 (after correction for multiple testing) was taken as signifying positive selection at the marked branches. In all tests performed in Datamonkey, the appropriate evolutionary model for each data set was directly estimated by the neighbor-joining method (NJ) on this web server.

### 2.5. Protein-Level Approach Tests

Positive selection was also examined at the protein level by adopting the method of McClellan and McCracken [32] in TreeSAAP [33], which takes into account the magnitude of the effect of the amino acid replacements on a predefined set of physicochemical properties. Following the recommendation by the authors, we considered changes with a magnitude ≥6 (*p* ≤ 0.01) as an indicator of the radical positive selection for a given physicochemical property. To obtain the topologies required for the analyses, each multiple sequence alignment (MSA) was used to construct an ML tree with the automatically inferred model and ultrafast bootstrap analysis implemented in IQ-TREE software [34,35,36] in its online version [37].

## 3. Results

### 3.1. Organization of Idgf Open Reading Frames and Genes

**Figure 1:**
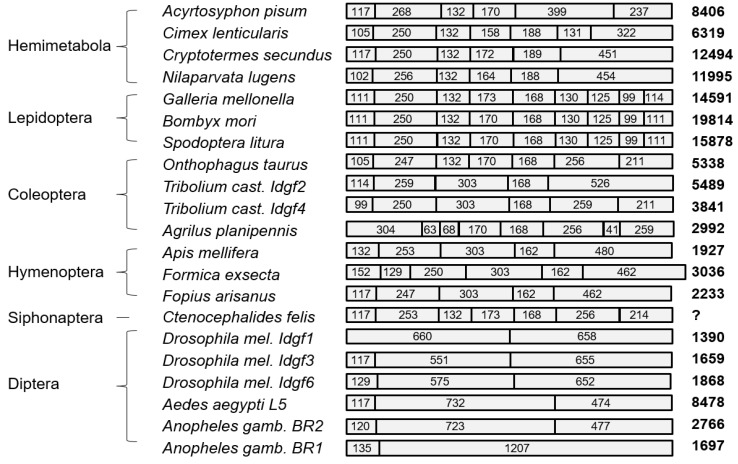
Comparison of exon arrangements of *Idgf* genes in representative insect species. Numbers within boxes indicate the length of individual exons. Lengths of the first and last exons do not include the untranslated leader and trailer sequences, respectively. Bold numbers on the right denote gene sizes without leader and trailer sequences (combined sizes of open reading frames (ORFs) and introns). GenBank accession numbers of the sequences and full names of insect species are shown in Table S1.

In order to characterize the *Idgf* gene family, we surveyed the publically available sequence data in the GenBank database. We retrieved genomic *Idgf* sequences from 17 species representing several orders of both holo- and hemimetabolous insects (Figure 1). Interestingly, the total size and overall organization of putative proteins were quite conserved, suggesting a structural constraint. Each IDGF protein contains a single putative carbohydrate binding site that exhibits sequence homology with chitinase catalytic domains. They also contain a conserved structure of the triose-phosphate isomerase barrel-fold of the 18 glycosyl hydrolase family, an IDGF-specific 24 extra amino acid residues between β-sheet 4 and α-helix 4, and an amino acid substitution of a key glutamic acid residue in the hydrolase active centre [8].

The total lengths of the open reading frames (ORFs) are in a narrow range, between 1281 and 1356 nucleotides (except for *Formica exsecta*, reaching 1458 bp, containing an extra exon sequence at the 5’ end). The observed differences in gene sizes, ranging from 1390 to 19,800 nucleotides, are largely driven by variations in the lengths of intronic sequences. As shown in Figure 1, the number of exons in *Idgf* genes varies from 2 to 9, with an average of 5.57 per gene. *Idgf* genes from the hemimetabolous insects contain 6–7 exons; similar numbers were also found in coleopteran and hymenopteran representatives and may signify the ancestral status. It seems that the arrangement of the first two exons is most conserved and that structural similarity gradually decreases towards the 3’ ends of the genes.

Interestingly, some other *Idgf* exons are spliced in a clade-specific way; in particular, the arrangement of nine exons among Lepidoptera seems to be well conserved. For example, the entire *Bombyx BmIdgf* gene contains nine exons and is almost 23 kb long [10]. The first introns in lepidopteran *Idgf* genes are the largest, spanning approximately 10 kb. In contrast, dipteran *Idgf* genes tend to be smaller, having 1 or 2 introns of short size (Table S1).

The comparison of known *Idgf* sequences also shows that insects may contain more *Idgf* paralogs depending on the evolutionary lineage. While a single *Idgf* gene seems to be present in hemimetabolous and some holometabolous species, multiple paralogs were detected in some holometabola, especially within the Schizophora group. For example, *D. melanogaster* contains six members of the *Idgf* family and *Lucilia cuprina* contains at least eight paralogs [38].

### 3.2. Phylogenetic Relationship of Idgfs

To elicit more information on the evolution of *Idgfs* in insects, we focused on Diptera and Lepidoptera, as they have both undergone profound changes in their exon-intron structure or contained more *Idgf* copies. We also generated data on *Idgfs* for additional representatives covering more primitive lepidopteran lineages, as well as Trichoptera, by sequencing cDNA libraries from 13 species of Lepidoptera as well as 5 species of Trichoptera, and identified a number of novel *Idgfs*. The total alignment was 1620 bp long and contained 145 *Idgf* sequences, with the number of codons ranging from 322 in *Idgf2* from *Glossina morsitans* to 474 in *Plodia interpunctella* (Figure S1).

**Figure 2:**
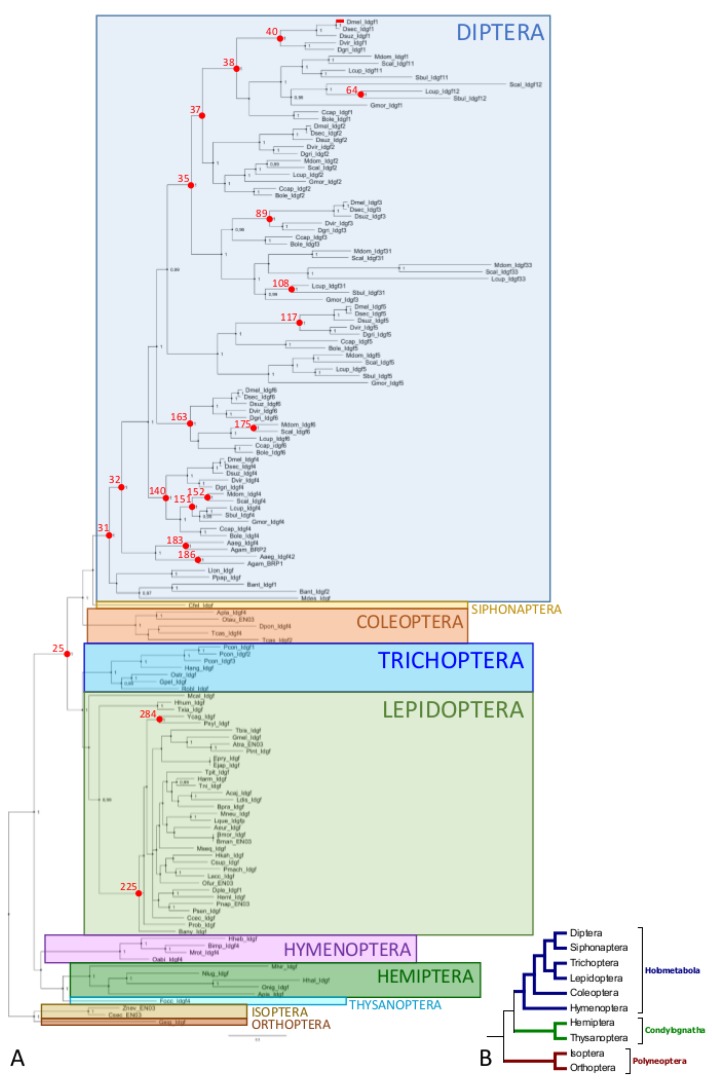
Relationships between the *Idgf* gene tree and species tree. (**A**) Phylogenetic tree of insect *Idgf* coding sequences reconstructed by the Bayesian inference (BI) method. Posterior probabilities higher than 0.95 are shown in black in well supported nodes. The abbreviations for terminal nodes are shown in Table S1. The nodes in which positive selection was detected by adaptive branch-site random effects likelihood (aBSREL) analysis are highlighted by red dots. Node numbers are depicted in red. Statistics and aBSREL results can be found in Table S2. (**B**) Phylogenetic relationship between examined insect orders, as suggested by Misof et al. [43].

We reconstructed the *Idgf* phylogenetic tree using minimum evolution, maximum likelihood, and Bayesian methods. All tested methods gave us similar results (compare Figure 2A with Figure S2A,B). We found that the phylogeny of *Idgfs* almost fully recovers the arrangement of examined species into accepted orders and families (except for the families Crambidae and Nymphalidae within Lepidoptera). The *Idgf* gene tree shows well resolved relationships among the main insect orders (compare Figure 2A with Figure 2B) with two exceptions. Namely, (i) the position of Hymenoptera is not with other Holometabola, as expected, and has very poor support (posterior probability of 0.56, aBayes value of 72, and 50% bootstrap). Notably, Coleoptera clustered together with Diptera and Siphonaptera with quite high support (posterior probability of 1, aBayes value of 99, and 70% bootstrap). (ii) The resolution of relationships among lepidopteran families, which can be influenced by rapid radiation in the evolutionary history of Lepidoptera.

The tree (Figure 2) indicates that the original ancestral state is the existence of a single copy of the *Idgf* gene in each insect species, supported by the representatives of hemimetabolous insects, as well as in Hymenoptera, Lepidoptera, and some primitive Diptera. *Idgfs* seem to be an insect-specific family, evolved from a common ancestor that preceded the separation of Polyneoptera, Condylognatha, and Holometabola. The oldest lineage in which we were able to detect *Idgfs* was that of polyneopteran insects, from the orders Orthoptera and Isoptera. Consistently, we did not detect *Idgfs* in Collembola, a sister group of insects.

The branching of the *Idgf* phylogenetic tree suggests that the *Idgf* genes might have evolved with asymmetric evolutionary rates, especially the duplicated genes in Diptera, which seem to have evolved quickly. It also seems that the *Idgfs* underwent several independent gene duplications: one in Trichoptera, one in Coleoptera, one in mosquitoes, and one in higher Diptera (Schizophora). The most extensive duplication occurred at the clade leading to higher Diptera (Calyptratae), with up to eight *Idgf* paralogs. 

### 3.3. Diversification of Idgfs and Estimation of Evolutionary Rates

To perform a simple comparison of how *Idgfs* have diverged from each other, we estimated their differences using p-distance. The overal mean p-distance was calculated using pairwise comparison and bootstrapping (10,000 replicates). The proportion (p) of nucleotide sites at which the compared sequence differed was calculated for individual paralogs of *Idgfs* in higher Diptera, as well as for a single ortholog in Lepidoptera and their sublineage Noctuoidea (represented by species from families Noctuidae, Nolidae, Arctiidae, Lymantriidae, and Thaumatopoeidae). To compare the diversification of the higher Diptera with the diversification of the lepidopteran clade, we chose the noctuoid branch, whose length of evolutionary history roughly corresponds to that of the Schizophora group. The Schizophora group experienced approximately 65 million years of evolution [39], similar to the 70 million years of the Noctuoidea group [40]. The entire lepidopteran clade evolved approximately 190 million years ago [40]. The results show that the *Idgf3* gene exhibits the highest variability within the Schizophora lineage, whereas *Idgf4* and *Idgf6* are the least distant from the ancestral gene. As also shown in Table 1, the individual *Idgf* paralogs in the Schizophora clade evolved faster than the single *Idgf* ortholog in the lepidopteran noctuid clade over a similar time frame.

### 3.4. Positive Selection Participated in Radiation of the Idgf Gene Family in Diptera

In order to investigate the patterns of possible selection in *Idgf* coding sequences, we used tests based on the observation that a nonsynonymous (or replacement) substitution in a protein-coding sequence is more likely to influence the fitness of an organism than a random synonymous substitution that leaves the amino acid sequence unchanged. The direction and strength of the presumed selection acting on a protein-coding DNA sequence can thus be estimated from the ratio of nonsynonymous (Ka) to synonymous (Ks) nucleotide substitutions (ω) calculated across a gene sample; ω < 1 denotes negative or purifying selection, ω > 1 indicates positive or diversifying selection, and ω = 1 points to neutral evolution [41,42].

First, we estimated ω values using the aBSREL method for the whole *Idgf* gene tree and searched for episodic positive selection across the whole phylogeny. We identified 21 nodes that are potentially under the influence of positive selection (compare Figure 2A with Table S2). They are mostly placed within Diptera and two nodes are present in Lepidoptera. We also detected positive selection at branching points leading to Schizophora *Idgf1*, *Idgf4*, and *Idgf6*, as well as *bacteria-responsive genes 1* and *2* (*AgBR1* and *2*) within Culicidae (see nodes 38, 163, 140, 183, and 186 in Figure 2A and Table S2). This can support a hypothesis that duplications of *Idgf* genes within Diptera may be accompanied by neofunctionalization and positive selection.

For further verification of positive selection, and because the tests for selection can be influenced by high divergence in a large scale phylogeny, we focused our subsequent analysis only on Diptera and Lepidoptera; we divided our examined sequences into subsets representing either individual *Idgf1-6* orthologs from Schizophora or *Idgfs* from Lepidoptera alone or in combination with their sister group Trichoptera. Subset analysis in aBSREL confirmed positive selection for the *Idgf4* clade (compare Figure S3D,H with Figure 2) as well as Lepidoptran clades Ditrysia and Yponomeutoidea (compare Figure S3G,H with Figure 2). Subsequent analysis with TreeSAAP localized several positively selected changes on the IDGF4 molecule that included a signal peptide, α1–α8 domains, and an insert between α7 and β7 domains known as αβ domain insertion (Table S3A). The majority of positively selected changes within Lepidoptera were localized to interdomain loops, including loops between β4 and α4, which distinguishes IDGFs from bona fide chitinases (Table S3B,C).

As the selection acting on the genes is a rather complex phenomenon, it is advisable to combine and corroborate results from several analyses for better data support. Following this approach, we used several state-of-the-art tests, each being suited for a particular type of selective forces. The web-based suite Datamonkey implements methods that allow positive or negative selection to be traced, either on sites or specific branches of the underlying phylogeny. Depending on the chosen method, selection is estimated as either episodic (acting only on a subset of sites or branches) or pervasive (acting on the whole phylogeny). Finally, we evaluated the potential impact of amino acid changes using TreeSAAP. The results were evaluated using a conservative approach and only sites suggested to be under selection by two or more methods were considered (Table 2). 

**Figure 3:**
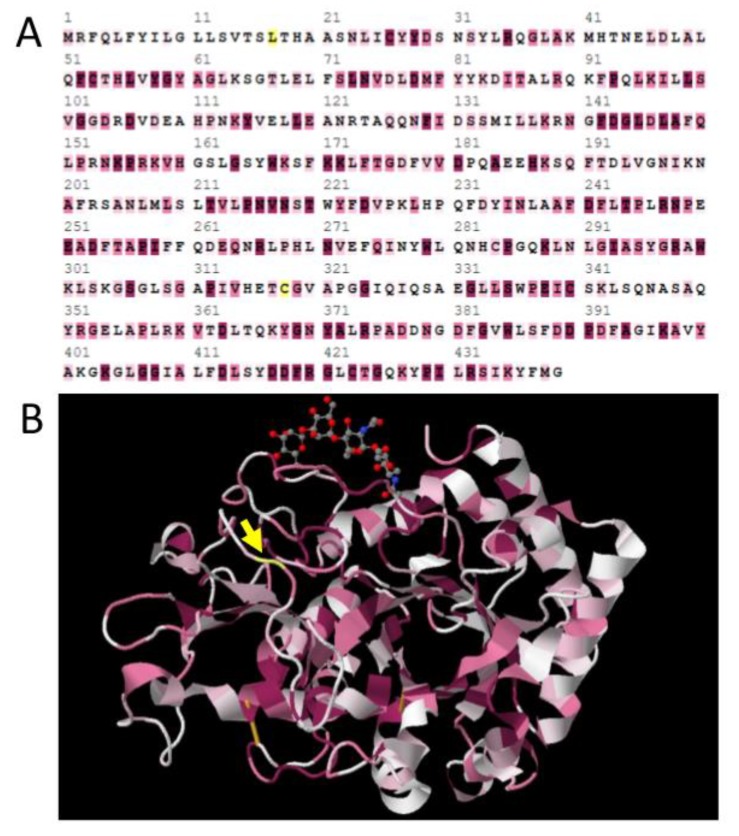
(**A**) Selection test results for the *Idgf1* gene from Schizophora mapped into the linear protein sequence of *Drosophila melanogaster* IDGF1, as evaluated by Selecton software. Letters highlighted in shades of purple represent the sites under purifying selection, and those in yellow are sites under positive selection. Only position 318 (codon 345 in the alignment of the *Idgf1* gene) could be confirmed by other methods. (**B**) Selecton results for the *Idgf1* gene from Schizophora mapped onto a prototypical 3D structure of IDGF protein [8]. The yellow arrow highlights position 318, which is under positive selection; shades of purple highlight the sites under purifying selection.

Positive selection was confirmed for the *Idgf1* branch, as has been previously suggested for aBSREL. In our analyses, position 345 of *Idgf1* was identified as positively selected by analyses including Selecton, MEME, Fel, FUBAR, and also TreeSAAP. Another positively selected site was detected at position 31 in the signal peptide of the *Idgf5* gene (Table 2). The Selecton results combined with a viewer for chemical structures, JMOL [44], allowed for these sites to be projected onto a 3D protein structure model, whereby potentially positively selected as well as negatively selected sites can be highlighted (Figure 3). As shown in Figure 3A, positively selected site 345 of the *Idgf1* gene is located in the αβ domain insertion between the domains α7 and β7. Selecton also revealed that many positions are quite conserved and evolved under purifying selection (because ω < 1) caused by selective constraints at codons.

### 3.5. Negative Selection Preserves Conserved Structure of the 18 Glycosyl Hydrolase Domain

We further compared the results from the Selecton, FEL, and FUBAR tests to identify the position under the influence of negative selection. We used a similar approach to that of the positive selection results and focused only on positions that were supported by multiple methods. Combined analysis revealed a high proportion of negatively selected positions, and we again detected differences between lepidopteran and dipteran sequences. Within Lepidoptera, we found that more than 80% of the *Idgf* molecule is under negative selection (see Table S4) and that positions under strong purifying selection were mostly localized in structurally important domains, β3–β8 (Table S5). However, proportions of negatively selected sites within Diptera were always lower (63%–75%) (Table S4). A RELAX test identified significant intensification of purifying selection within the alignment of lepidopteran *Idgfs* (Table 3). No significant results were detected for *Idgf1–6* from Diptera. 

## 4. Discussion

Advances in sequencing technologies and the wide use of RNAi-based reverse genetics methods outside traditional model organisms allow not only for a comparative functional analysis between organisms, but also to search for the evolutionary origins of various functions to be traced. Functional analyses of *Idgfs* in different insect species suggest that the importance of these genes might grow during insect evolution. While no obvious phenotypes were observed after the knockdown of *Idgf* orthologs in two hemimetabolous species (*N. lugens* and *P. solenopsis*), the strongest effects of knocking down various *Idgf* genes were observed in Diptera (Table 4). Several *Idgf* genes in *D. melanogaster* and *T. castaneum* showed cuticular defects and molting problems similar to that caused by the knockdown of enzymatically active chitinases. This suggests that these genes and their expressed proteins are involved in the maintenance of the cuticle or extracellular-matrix (ECM). On the other hand, some paralogs in Diptera show additional phenotypes, including responses to injury, support of cell growth, and maintenance of the barrier against infection; the mosquito homolog is even able to influence the mammalian host immune response (Table 4). Further functional studies are needed to clarify the correlation between sequence level evolutionary changes and *Idgf* functions. 

Our results support the idea that *Idgf* genes are monophyletic, having evolved in hemimetabolous insects from chitinases that lost their enzymatic function. IDGF proteins are still able to bind some carbohydrates. Herein, we show that the different proteins in the IDGF family evolved with varying speeds among insects. Phylogenetic analyses suggested gene duplication and accelerated diversification of *Idgfs* in Diptera, which is accompanied by signs of positive selection. In contrast, in lepidopteran genomes, *Idgf* genes exist as a single copy; they are more conserved and characterized by strong negative selection. We suggest that the increased rate of divergence in Diptera accompanied by positive selection is the result of the gain of a new function. This function might be connected with innate immune responses. Alternatively, multifunctional proteins might duplicate and specialize for individual functions. It may be possible that the expansion of growth factors reflects the fast development of higher Diptera. A similar expansion can be observed in the adenosine deaminase growth factors (ADGFs), which also have six members in *D. melanogaster* [45].

Responses to injury require fast production of large amounts of gene products. It may be important that *Drosophila Idgf* genes are short and have a simple exon-intron structure, which would be consistent with the idea of fast responses. In contrast, lepidopteran *Idgf* genes are large (around 20 kb) and tend to have nine exons, which would delay the responses (“intron delay” hypothesis, [46]). Alternatively, there might be a general tendency for an increase of gene sizes in Lepidoptera. Numerous lepidopteran genes have been shown to have a complex exon-intron structure, which contrasts with the simple structure of their dipteran orthologs [47]. The size differences thus may be connected to genome sizes, which are larger for Lepidoptera.

In the current study, we focused on selection tests based on the whole insect phylogeny. We found that more than 80% of the IDGF molecule is under negative selection within Lepidoptera, with selected sites mostly localized in conserved domains needed for stability of the (βα)_8_-barrel structure. A similar extent of negative selection was previously observed among lepidopteran fibroin light chain genes [57], which encode the most conserved silk proteins. We also found that some regions in dipteran *Idgf1* and *Idgf4* genes evolved under positive selection. Selection on *Idgf1* has already been investigated using intraspecific data on *Idgf1* and *Idgf3* from natural populations of *D. melanogaster* [12,58]. In those studies, patterns of polymorphism suggested that *Idgf3* is neutral, consistent with our new data. However, *Idgf1* showed some signs of balancing selection and further investigations will be needed to clarify the exact pattern of its evolution.

Our data on IDGF1 protein show that positively selected changes are localized into an α7β7 domain insertion and can influence the polarity and proximity of the amino acid to the protein surface. Interestingly, four positively selected changes in the α7β7 domain insertion were also detected in IDGF4 by combination of the aBSREL and TreeSAAP methods. One of the IDGF4 positions was localized adjacent to the position detected at IDGF1 (Figure S4). The αβ domain insertion between the α7 helix and β7 strand of chitinase was previously studied using bioinformatic methods; it has been suggested that it influences the stability of the binding pocket, as well as substrate specificity and affinity [59]. Another study on *Serratia marcescens* chitinase experimentally confirmed the effect of α7β7 domain insertion on substrate binding and showed that certain changes in the αβ domain insertion can also affect thermal stability of the whole protein [60]. Further experiments are needed to confirm the importance of α7β7 domain insertion in IDGFs.

## 5. Conclusions

IDGFs are insect-specific proteins that evolved from a common ancestor that preceded the separation of Polyneoptera, Condylognatha, and Holometabola. 

The original ancestral state is a single copy of the *Idgf* gene per insect species, which is supported by representatives of hemimetabolous insects, as well as in Hymenoptera, Lepidoptera, and some primitive Diptera.

The duplicated *Idgf* genes in Diptera evolve faster than the unique ones in other insects. 

We suggest that the increased rate of *Idgf* divergence in Diptera accompanied by positive selection is the result of the gain of a new function.

We found signs of positive selection in dipteran *Idgf1* and *Idgf4* genes. Most of the positive selection changes in IDGF1 and IDGF4 proteins are localized into the α7β7 domain insertion implicated in substrate binding and thermal stability of related chitinase proteins.

## Figures and Tables

**Table 1:** Diversification of *Idgf* coding sequences within higher Diptera (Schizophora) and Lepidoptera (Noctuoidea).

Gene and Clade	Overall Mean *p*-Distance	S.E.
Schizophora * *Idgf1*	0.397	0.007
Schizophora * *Idgf2*	0.311	0.007
Schizophora * *Idgf3*	0.418	0.007
Schizophora * *Idgf4*	0.261	0.007
Schizophora * *Idgf5*	0.383	0.008
Schizophora * *Idgf6*	0.270	0.007
Lepidoptera ** *Idgf*	0.293	0.007
Noctuoidea *** *Idgf*	0.229	0.008

Number of species examined: * Schizophora (12), ** Lepidoptera (36), *** Noctuoidea (6).

**Table 2:** Sites under positive selection according to five different methods (see text for detailed description).

Gene and Clade		Datamonkey Selection Tests				TreeSAAP	
	Site	Selecton	MEME	FEL	FUBAR	Property	Direction
Schizophora *Idgf1*	345	x	x	x	x	B Br p	neg pos pos
Schizophora *Idgf5*	31	x	x	x	x	p Ra P	pos neg pos

Abbreviations: x = positive test result; B = Beta structure tendencies; Br = buriedness (a proximity of the amino acid to the surface of the protein); p = polarity; Ra = solvent accessible reduction ratio; P = turn tendencies; MEME = mixed effects model of evolution; FUBAR = fast unconstrained Bayesian approximation; FEL = fixed effects likelihood.

**Table 3:** RELAX test results.

Gene and Clade	RELAX		
	k	p	LR
Schizophora *Idgf1*	1.8	0.085 ^ns^	2.97
Schizophora *Idgf2*	0.38	1.000 ^ns^	−4723.04
	1.25	1.000 ^ns^	−144.45
Schizophora *Idgf3*	1.01	1.000 ^ns^	−0.04
Schizophora *Idgf4*	4.36	1.000 ^ns^	−3.12
Schizophora *Idgf5*	1.00	0.953 ^ns^	0.00
Schizophora *Idgf6*	1.74	0.214 ^ns^	1.55
Lepidoptera	1.21	0.004 **	8.11

k (parameter; purifying selection is intensified for k > 1 or relaxed for k < 1 compared with background branches); p (probability; ns—non significant, ** *p* < 0.01); LR (likelihood ratio).

**Table 4:** Functional effects of various imaginal disc growth factors (IDGFs). ECM, extracellular-matrix.

Species	Effect	Reference
*Nilaparvata lugens*	*NiIdgf* knockdown has no effect on morphology and survival	[48]
*Phenacoccus solenopsis*	*PsIdgf* knockdown has no effect on morphology and survival	[49]
*Bombyx mori*	BmIDGF is modulated in response to nutritional conditions	[50]
*Bombyx mori*	BmIDGF is induced by apoptosis or by ecdysone	[51]
*Pieris rapae*	IDGF is not affected by parasitization and polydnavirus infection	[52]
*Mamestra brassicae*	MbIDGF supports growth of two lepidopteran cell lines	[53]
*Manduca sexta*	In the presence of HAIP, cells in culture do not form aggregates	[9]
*Tribolium castaneum*	*TcIdgf4* is involved in the molting process	[54]
*Tribolium castaneum*	*TcIdgf2* knockdown has no phenotypic effect	[54]
*Anopheles gambiae*	AgBR1 and 2 are immune responsive proteins to bacteria	[6]
*Aedes aegypti*	AgBR1 influences mammalian host immune response	[7]
*Bactrocera dorsalis*	*Idgf4* knockdown decreased larval survival under high temperature	[55]
*Drosophila melanogaster*	*Idfg6* knockdown causes severe cuticular defects	[5]
*Drosophila melanogaster*	IDGF1,3,4,5, and 6 are required for chitin-ECM formation	[5]
*Drosophila melanogaster*	IDGF2 is induced by injury, supports growth of Cl.8 cells in vitro	[3]
*Drosophila melanogaster*	IDGF3 is needed for hemolymph clotting	[4]
*Drosophila melanogaster*	IDGF1 and 3 are involved in response to septic injury	[56]

HAIP—hemocyte aggregation inhibitor protein.

## Supplementary Materials

The following are available online at https://www.mdpi.com/2075-4450/10/10/365/s1, Figure S1. Multiple sequence alignment of 145 *Idgf* coding sequences; Figure S2. Phylogenetic analysis of *Idgf* coding sequences; Figure S3. Maximum likelihood phylogenetic trees with highlighted nodes under positive selection as detected by aBSREL; Figure S4. Alignment of IDGFs with the indicated structural motifs and positively selected sites in IDGF1 and 4; Table S1. List of sequences used in this study; Table S2. aBSREL test results and statistics for positively selected nodes; Table S3. Positively selected sites and change in amino acid properties detected by TreeSAAP analysis; Table S4. Proportions of positions under purifying selection; Table S5. List of negatively selected positions from lepidopteran IDGF alignment.
