# Supplementary material for: Expansion of Imaginal Disc Growth Factor Gene Family in Diptera Reflects the Evolution of Novel Functions
Source: Insects. 2019 Oct 20;10(10):365. doi: 10.3390/insects10100365 (PMC6835396; doi:10.3390/insects10100365)
Supplement: Supplementary file 1 [file insects-10-00365-s001.pdf]

Supplementary material

Figure S1. Multiple sequence alignment of 145 *Idgfs* coding sequences across insect taxa, which we used for subsequent phylogenetic analysis and selection tests. Species list with abbreviations is in Table S1.

Figure S2. Phylogenetic analysis of *Idgf* coding sequences. **(A)** Minimum Evolution (ME) tree, brach support tested by bootstrap method with 10000 replicates (only values above 50 are shown next to well supported nodes). **(B)** Maximum-Likelihood tree, found by Neutral Network Intelligence (NNI) toolkit, aBayes values higher than 50 shown next to well supported nodes. **(C)** Maximum-Likelihood tree, found by Subtree-Pruning-Regrafting (SPR) search algorithm, 10 replicates, aBayes values higher than 50 shown next to well supported nodes.

Figure S3. Maximum likelihood phylogenetic trees with highlighted nodes under positive selection as detected by aBSREL (dots). Nodes dyed in red confirmed results from aBSREL analysis on large phylogeny (compare with Fig. 2), black nodes are unique and they were not confirmed by other analysis. **(A)** Phylogenetic tree of *Idgf1* from Schizophora, *Idgf* sequences from *M. destructor*, *A.gambiae* and *C. felis* were used as outgroups. **(B)** Phylogenetic tree of *Idgf2* from Schizophora, *Idgf* sequences from *M. destructor*, *A.gambiae* and *C. felis* were used as outgroups. **(C)** Phylogenetic tree of *Idgf3* from Schizophora, Idgf sequences from *M. destructor*, *A.gambiae* and *C. felis* were used as outgroups. **(D)** Phylogenetic tree of *Idgf4* from Schizophora, *Idgf* sequences from *M. destructor*, *A.gambiae* and *C. felis* were used as outgroups. **(E)** Phylogenetic tree of *Idgf5* from Schizophora, *Idgf* sequences from *M. destructor*, *A.gambiae* and *C. felis* were used as outgroups. **(F)** Phylogenetic tree of *Idgf6* from Schizophora, *Idgf* sequences from *M. destructor*, *A.gambiae* and *C. felis* were used as outgroups. **(G)** Phylogenetic tree of lepidopteran and trichopteran *Idgf* sequences with *C.felis*, *A. gambiae* and *D. melanogaster Idgf3* as outgroups. **(H)** Combined tree of *Idgf* sequences from Diptera (only *Idgf4* was taken as representative for Schizophora), Siphonaptera (*C. felis*), Trichoptera and Lepidoptera.

Figure S4. Protein alignment of IDGFs from *D. melanogaster*, *A. gambiae*, **O. striata**, *H. humuli*, *G. mellonella* and *B.mori* with highlighted α-helix, β-sheet structure. Modified catalytic binding site is shown (black arrow), positively selected positions localized within α7β7 domain insertion displayed in red rounds.

Table S1. List of sequences used in this study.

Table S2. aBSREL test results and statistics for positively selected nodes highlighted in Fig.2 phylogeny. B – optimized branch length, LRT – likelihood-ratio test, $\omega > 1$ (in red) – positive selection, $\omega < 1$ (in purple) – negative selection, $\omega = 1$ – neutral evolution.

Table S3. Positively selected sites and change in amino acid properties detected by TreeSAAP **analysis** in accordance with aBSREL result for Schizophora Idgf4 node **(A)**, Lepidoptera node **(B)** and Yponomeutoidea node **(C)**.

Table S4. Proportions of positions under purifying selection as detected by Selecton, FEL and FUBAR tests.

Table S5. List of negatively selected positions from Lepidoptera alignment mapped to *Hepialus humuli Idgf* sequence with statistics from Selecton, FEL and FUBAR tests.

Figure S1

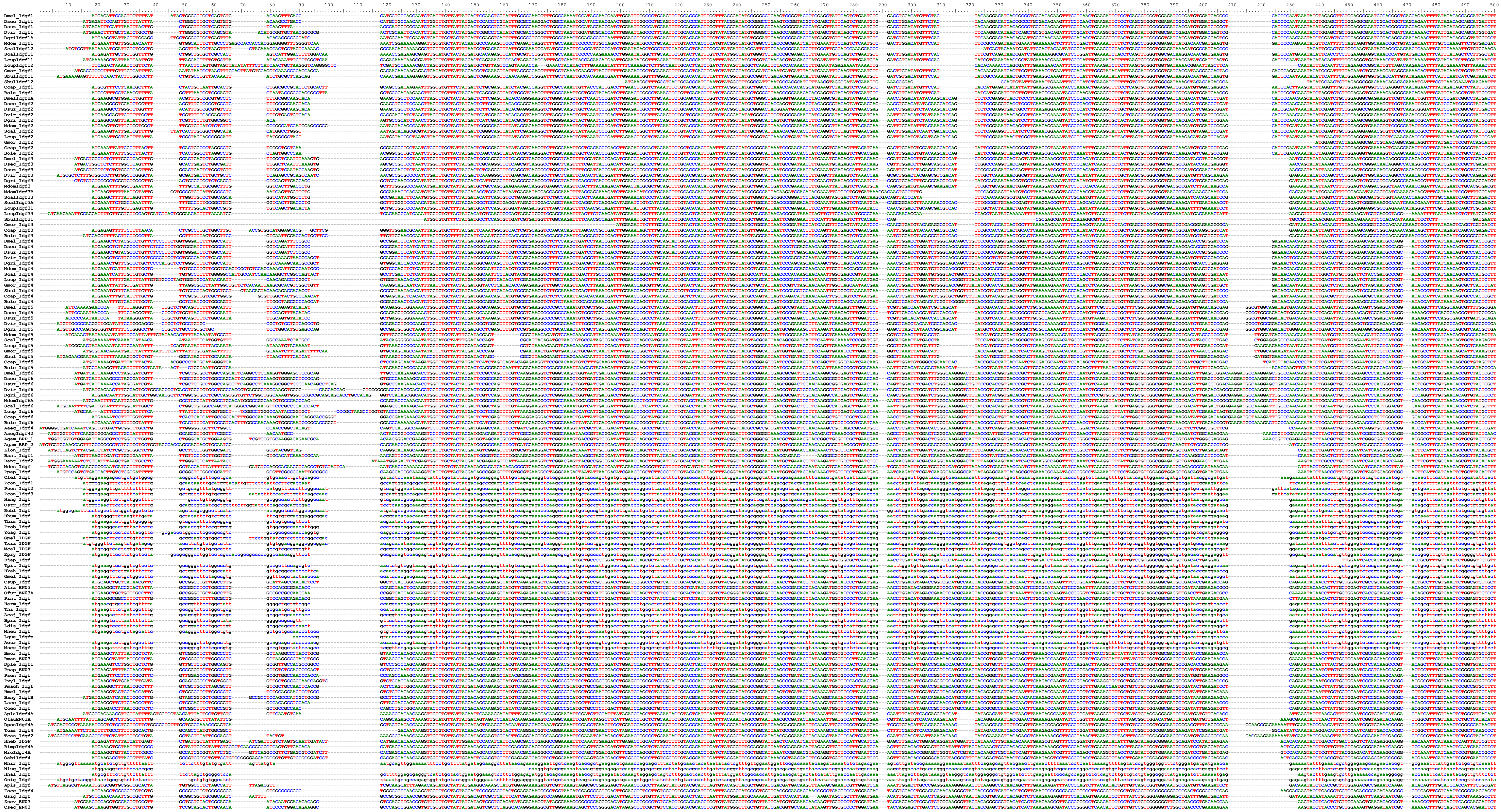

Figure S1

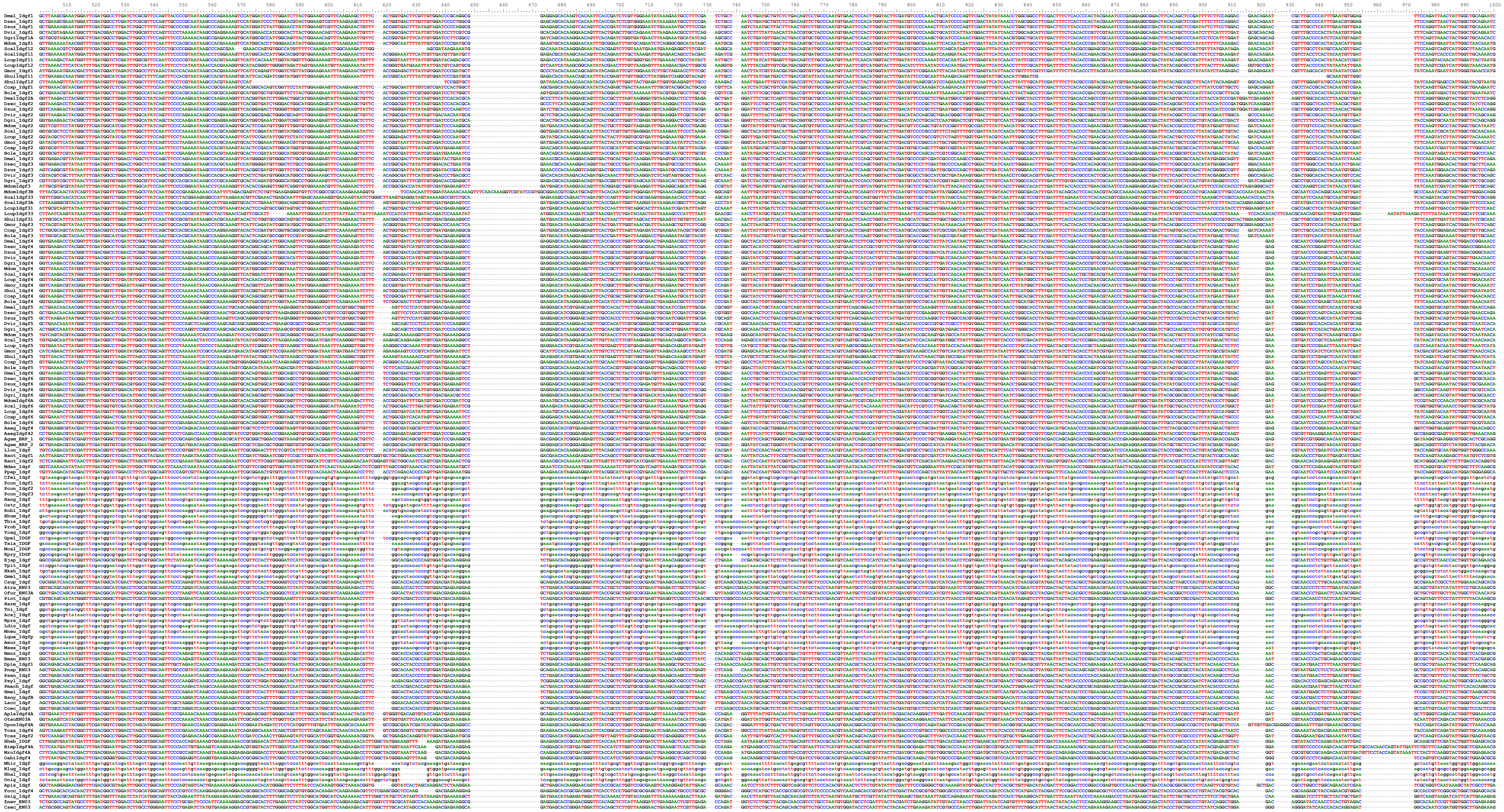

Figure S1

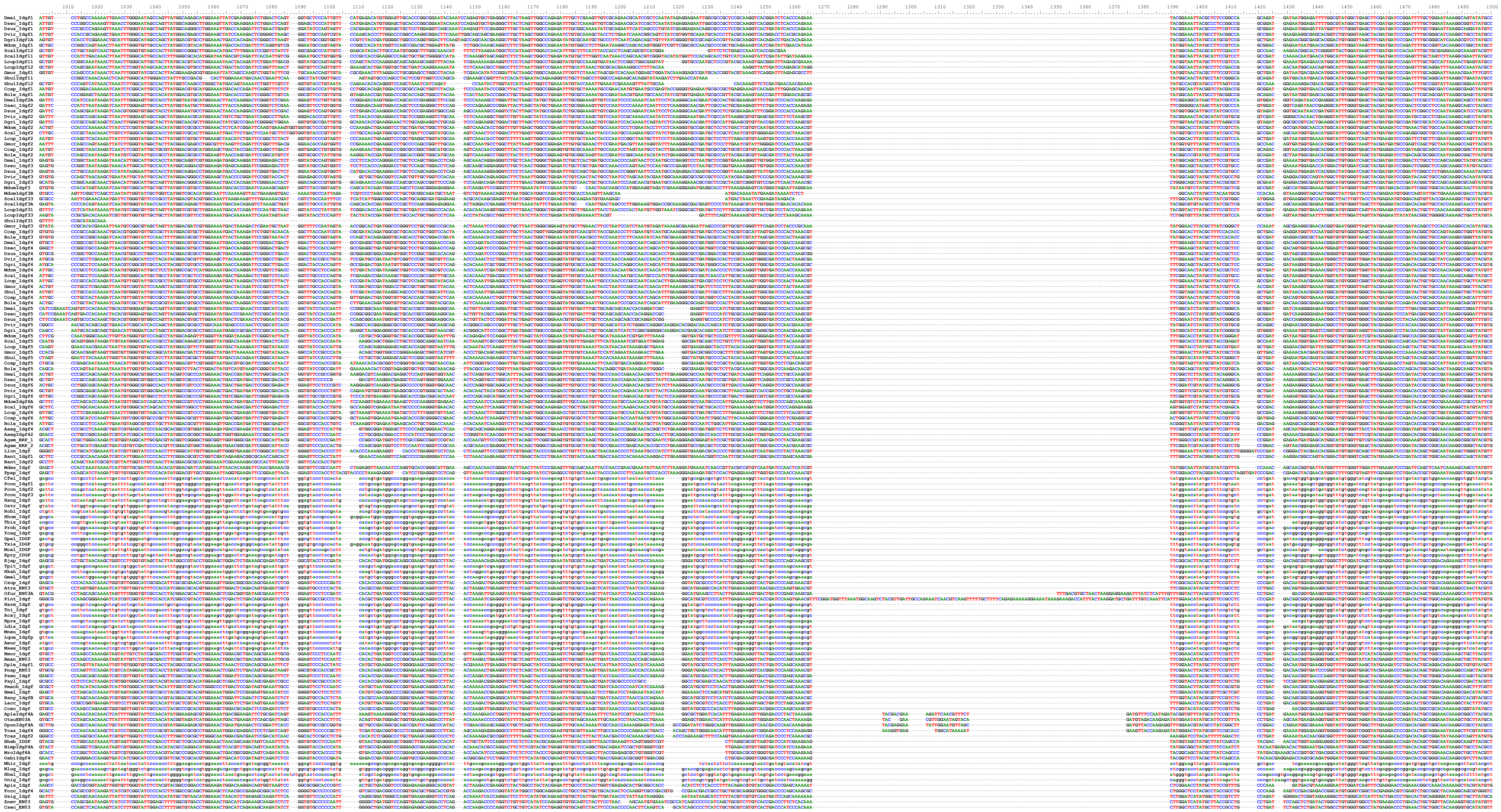

Figure S1

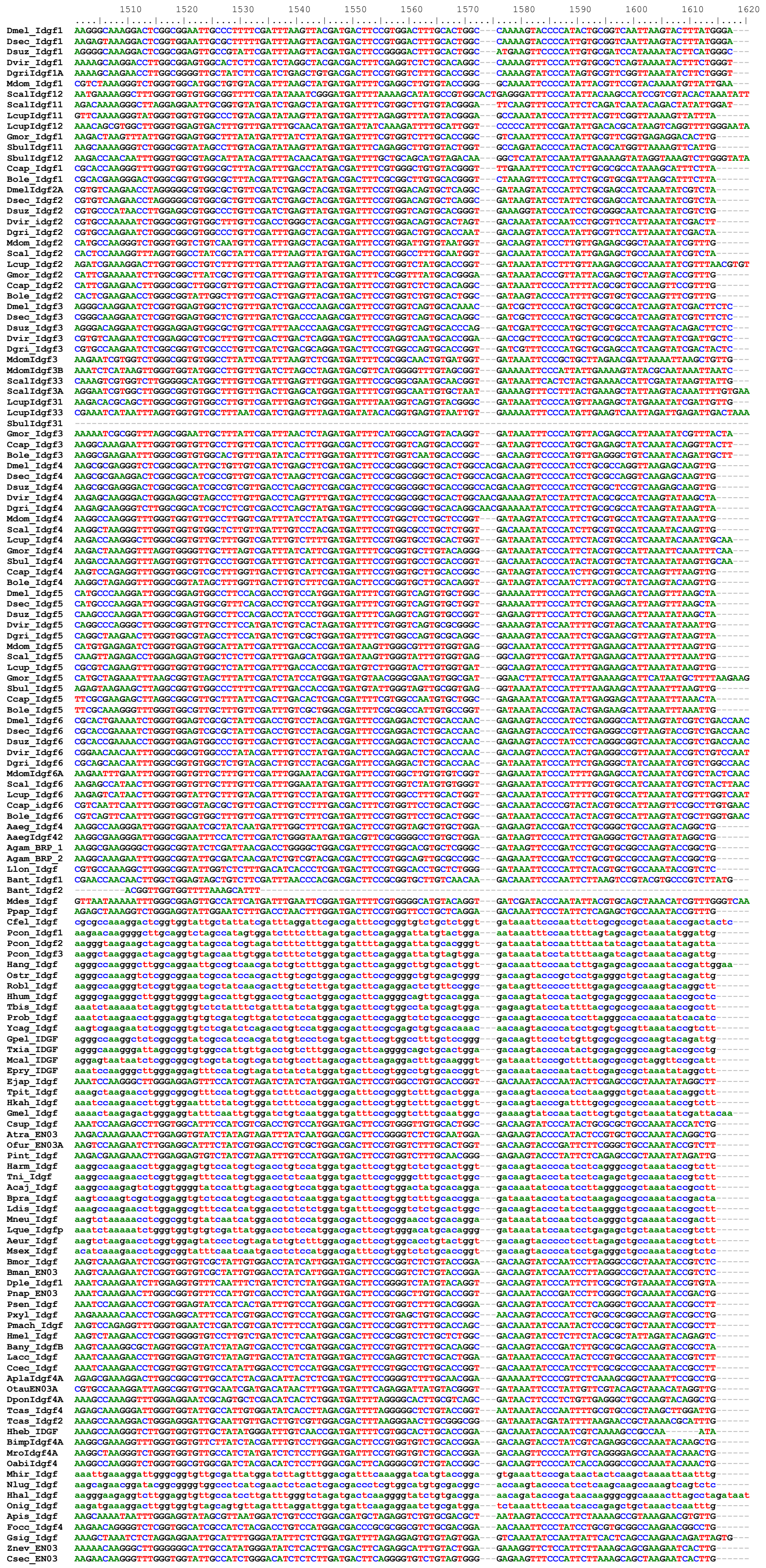

Figure S2A

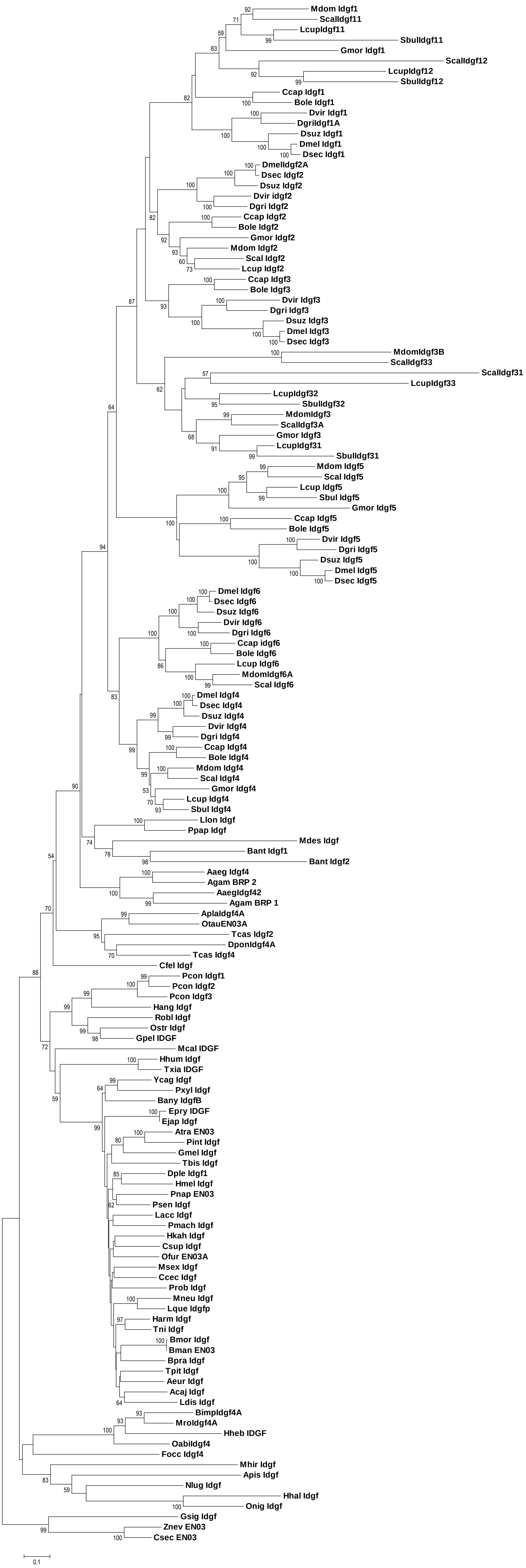

Mdom Idgf1
ScalIdgf11
LcupIdgf11
SbulIdgf11
Gmor Idgf1
ScalIdgf12
LcupIdgf12
SbulIdgf12
Ccap Idgf1
Bole Idgf1
Dvir Idgf1
DgriIdgf1A
Dsuz Idgf1
Dmel Idgf1
Dsec Idgf1
DmelIdgf2A
Dsec Idgf2
Dsuz Idgf2
Dvir idgf2
Dgri Idgf2
Ccap Idgf2
Bole Idgf2
Gmor Idgf2
Mdom Idgf2
Scal Idgf2
Lcup Idgf2
Ccap Idgf3
Bole Idgf3
Dvir Idgf3
Dgri Idgf3
Dsuz Idgf3
Dmel Idgf3
Dsec Idgf3
MdomIdgf3B
ScalIdgf33
ScalIdgf31
LcupIdgf33
LcupIdgf32
SbulIdgf32
MdomIdgf3
ScalIdgf3A
Gmor Idgf3
LcupIdgf31
SbulIdgf31
Mdom Idgf5
Scal Idgf5
Lcup Idgf5
Sbul Idgf5
Gmor Idgf5
Ccap Idgf5
Bole Idgf5
Dvir Idgf5
Dgri Idgf5
Dsuz Idgf5
Dmel Idgf5
Dsec Idgf5
Dmel Idgf6
Dsec Idgf6
Dsuz Idgf6
Dvir Idgf6
Dgri Idgf6
Ccap idgf6
Bole Idgf6
Lcup Idgf6
MdomIdgf6A
Scal Idgf6
Dmel Idgf4
Dsec Idgf4
Dsuz Idgf4
Dvir Idgf4
Dgri Idgf4
Ccap Idgf4
Bole Idgf4
Mdom Idgf4
Scal Idgf4
Gmor Idgf4
Lcup Idgf4
Sbul Idgf4
Llon Idgf
Ppap Idgf
Mdes Idgf
Bant Idgf1
Bant Idgf2
Aaeg Idgf4
Agam BRP 2
AaegIdgf42
Agam BRP 1
AplaIdgf4A
OtauEN03A
Tcas Idgf2
DponIdgf4A
Tcas Idgf4
Cfel Idgf
Pcon Idgf1
Pcon Idgf2
Pcon Idgf3
Hang Idgf
Robl Idgf
Ostr Idgf
Gpel IDGF
Mcal IDGF
Hhum Idgf
Txia IDGF
Ycag Idgf
Pxyl Idgf
Bany IdgfB
Epry IDGF
Ejap Idgf
Atra EN03
Pint Idgf
Gmel Idgf
Tbis Idgf
Dple Idgf1
Hmel Idgf
Pnap EN03
Psen Idgf
Lacc Idgf
Pmach Idgf
Hkah Idgf
Csup Idgf
Ofur EN03A
Msex Idgf
Ccec Idgf
Prob Idgf
Mneu Idgf
Lque Idgfp
Harm Idgf
Tni Idgf
Bmor Idgf
Bman EN03
Bpra Idgf
Tpit Idgf
Aeur Idgf
Acaj Idgf
Ldis Idgf
BimpIdgf4A
MroIdgf4A
Hheb IDGF
OabiIdgf4
Focc Idgf4
Mhir Idgf
Apis Idgf
Nlug Idgf
Hhal Idgf
Onig Idgf
Gsig Idgf
Znev EN03
Csec EN03
0.1

A

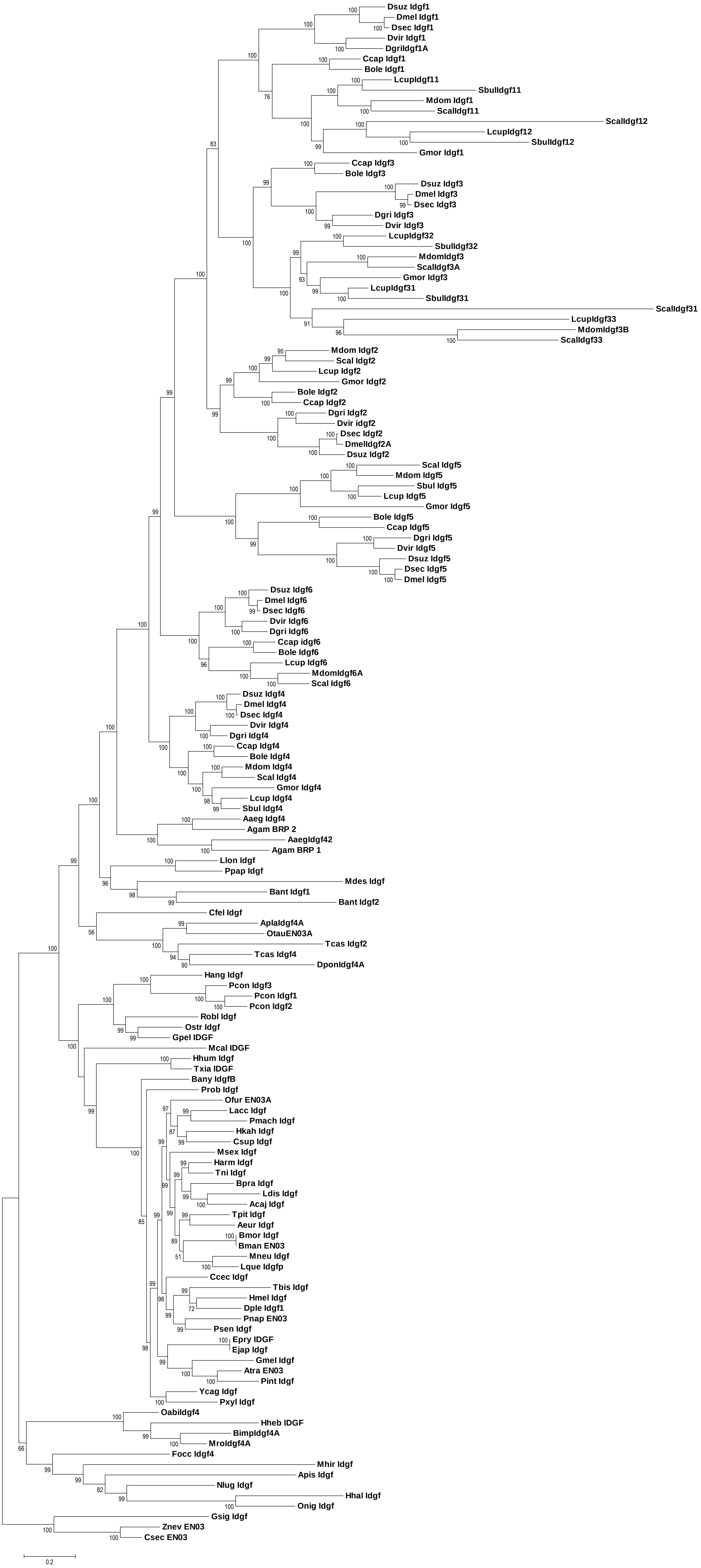
Dsuz Idgf1
Dmel Idgf1
Dsec Idgf1
Dvir Idgf1
DgriIdgf1A
Ccap Idgf1
Bole Idgf1
LcupIdgf11
SbulIdgf11
Mdom Idgf1
ScalIdgf11
ScalIdgf12
LcupIdgf12
SbulIdgf12
Gmor Idgf1
Ccap Idgf3
Bole Idgf3
Dsuz Idgf3
Dmel Idgf3
Dsec Idgf3
Dgri Idgf3
Dvir Idgf3
LcupIdgf32
SbulIdgf32
MdomIdgf3
ScalIdgf3A
Gmor Idgf3
LcupIdgf31
SbulIdgf31
ScalIdgf31
LcupIdgf33
MdomIdgf3B
ScalIdgf33
Mdom Idgf2
Scal Idgf2
Lcup Idgf2
Gmor Idgf2
Bole Idgf2
Ccap Idgf2
Dgri Idgf2
Dvir idgf2
Dsec Idgf2
DmelIdgf2A
Dsuz Idgf2
Scal Idgf5
Mdom Idgf5
Sbul Idgf5
Lcup Idgf5
Gmor Idgf5
Bole Idgf5
Ccap Idgf5
Dgri Idgf5
Dvir Idgf5
Dsuz Idgf5
Dsec Idgf5
Dmel Idgf5
Dsuz Idgf6
Dmel Idgf6
Dsec Idgf6
Dvir Idgf6
Dgri Idgf6
Ccap idgf6
Bole Idgf6
Lcup Idgf6
MdomIdgf6A
Scal Idgf6
Dsuz Idgf4
Dmel Idgf4
Dsec Idgf4
Dvir Idgf4
Dgri Idgf4
Ccap Idgf4
Bole Idgf4
Mdom Idgf4
Scal Idgf4
Gmor Idgf4
Lcup Idgf4
Sbul Idgf4
Aaeg Idgf4
Agam BRP 2
AaegIdgf42
Agam BRP 1
Llon Idgf
Ppap Idgf
Mdes Idgf
Bant Idgf1
Bant Idgf2
Cfel Idgf
AplaIdgf4A
OtauEN03A
Tcas Idgf2
Tcas Idgf4
DponIdgf4A
Hang Idgf
Pcon Idgf3
Pcon Idgf1
Pcon Idgf2
Robl Idgf
Ostr Idgf
Gpel IDGF
Mcal IDGF
Hhum Idgf
Txia IDGF
Bany IdgfB
Prob Idgf
Ofur EN03A
Lacc Idgf
Pmach Idgf
Hkah Idgf
Csup Idgf
Msex Idgf
Harm Idgf
Tni Idgf
Bpra Idgf
Ldis Idgf
Acaj Idgf
Tpit Idgf
Aeur Idgf
Bmor Idgf
Bman EN03
Mneu Idgf
Lque Idgfp
Ccec Idgf
Tbis Idgf
Hmel Idgf
Dple Idgf1
Pnap EN03
Psen Idgf
Epry IDGF
Ejap Idgf
Gmel Idgf
Atra EN03
Pint Idgf
Ycag Idgf
Pxyl Idgf
OabiIdgf4
Hheb IDGF
BimpIdgf4A
MroIdgf4A
Focc Idgf4
Mhir Idgf
Apis Idgf
Nlug Idgf
Hhal Idgf
Onig Idgf
Gsig Idgf
Znev EN03
Csec EN03
0.2

B

Figure S2C

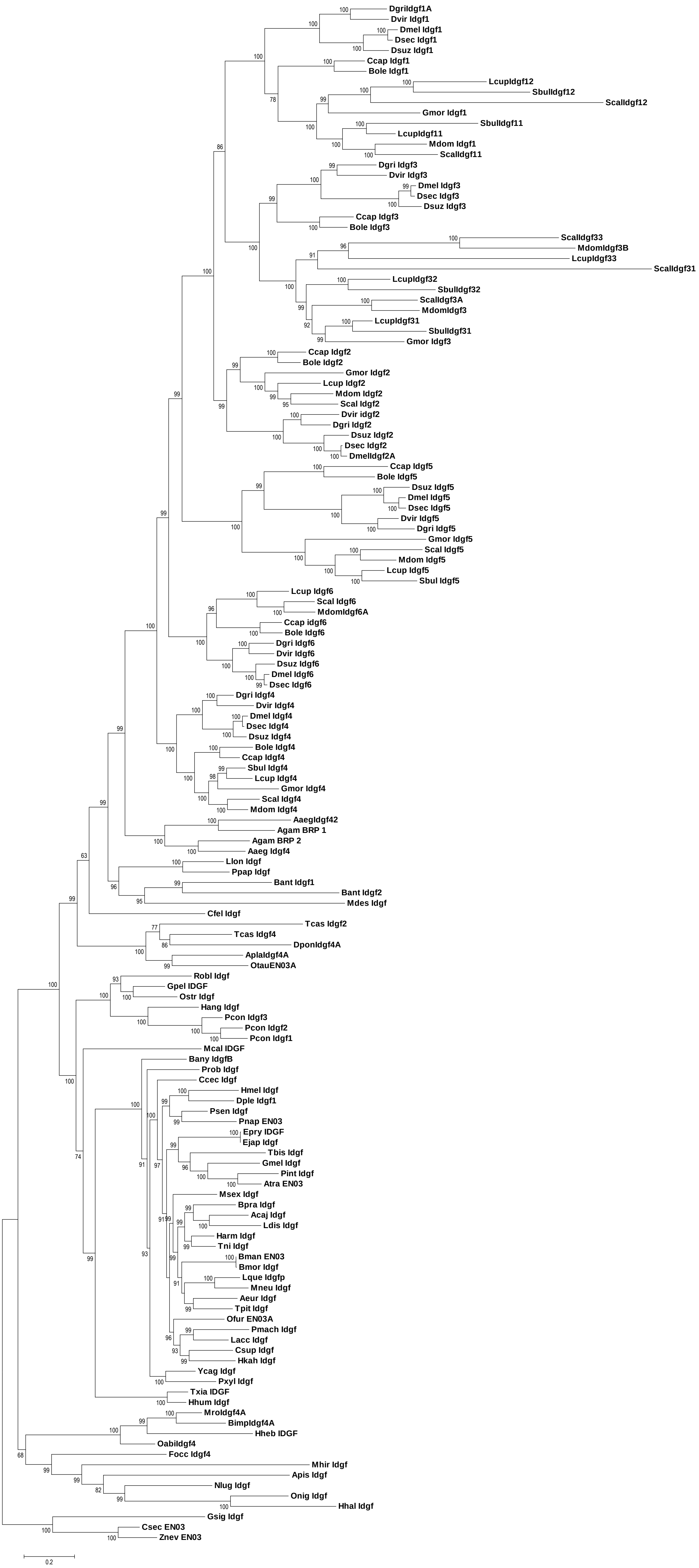
DgriIdgf1A
Dvir Idgf1
Dmel Idgf1
Dsec Idgf1
Dsuz Idgf1
Ccap Idgf1
Bole Idgf1
LcupIdgf12
SbulIdgf12
ScalIdgf12
Gmor Idgf1
SbulIdgf11
LcupIdgf11
Mdom Idgf1
ScalIdgf11
Dgri Idgf3
Dvir Idgf3
Dmel Idgf3
Dsec Idgf3
Dsuz Idgf3
Ccap Idgf3
Bole Idgf3
ScalIdgf33
MdomIdgf3B
LcupIdgf33
ScalIdgf31
LcupIdgf32
SbulIdgf32
ScalIdgf3A
MdomIdgf3
LcupIdgf31
SbulIdgf31
Gmor Idgf3
Ccap Idgf2
Bole Idgf2
Gmor Idgf2
Lcup Idgf2
Mdom Idgf2
Scal Idgf2
Dvir idgf2
Dgri Idgf2
Dsuz Idgf2
Dsec Idgf2
DmelIdgf2A
Ccap Idgf5
Bole Idgf5
Dsuz Idgf5
Dmel Idgf5
Dsec Idgf5
Dvir Idgf5
Dgri Idgf5
Gmor Idgf5
Scal Idgf5
Mdom Idgf5
Lcup Idgf5
Sbul Idgf5
Lcup Idgf6
Scal Idgf6
MdomIdgf6A
Ccap idgf6
Bole Idgf6
Dgri Idgf6
Dvir Idgf6
Dsuz Idgf6
Dmel Idgf6
Dsec Idgf6
Dgri Idgf4
Dvir Idgf4
Dmel Idgf4
Dsec Idgf4
Dsuz Idgf4
Bole Idgf4
Ccap Idgf4
Sbul Idgf4
Lcup Idgf4
Gmor Idgf4
Scal Idgf4
Mdom Idgf4
AaegIdgf42
Agam BRP 1
Agam BRP 2
Aaeg Idgf4
Llon Idgf
Ppap Idgf
Bant Idgf1
Bant Idgf2
Mdes Idgf
Cfel Idgf
Tcas Idgf2
Tcas Idgf4
DponIdgf4A
AplaIdgf4A
OtauEN03A
Robl Idgf
Gpel IDGF
Ostr Idgf
Hang Idgf
Pcon Idgf3
Pcon Idgf2
Pcon Idgf1
Mcal IDGF
Bany IdgfB
Prob Idgf
Ccec Idgf
Hmel Idgf
Dple Idgf1
Psen Idgf
Pnap EN03
Epry IDGF
Ejap Idgf
Tbis Idgf
Gmel Idgf
Pint Idgf
Atra EN03
Msex Idgf
Bpra Idgf
Acaj Idgf
Ldis Idgf
Harm Idgf
Tni Idgf
Bman EN03
Bmor Idgf
Lque Idgfp
Mneu Idgf
Aeur Idgf
Tpit Idgf
Ofur EN03A
Pmach Idgf
Lacc Idgf
Csup Idgf
Hkah Idgf
Ycag Idgf
Pxyl Idgf
Txia IDGF
Hhum Idgf
MroIdgf4A
BimpIdgf4A
Hheb IDGF
OabiIdgf4
Focc Idgf4
Mhir Idgf
Apis Idgf
Nlug Idgf
Onig Idgf
Hhal Idgf
Gsig Idgf
Csec EN03
Znev EN03
0.2

C

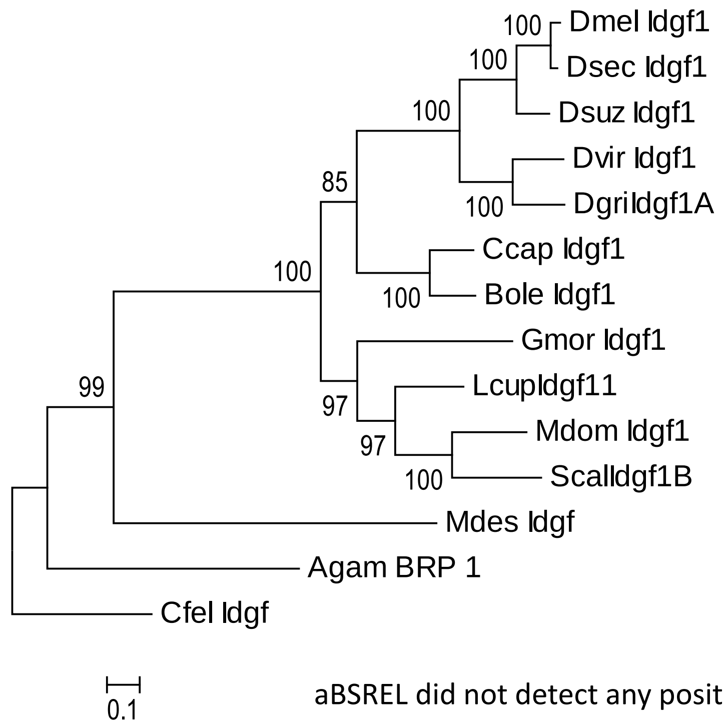

A aBSREL did not detect any positively selected nodes

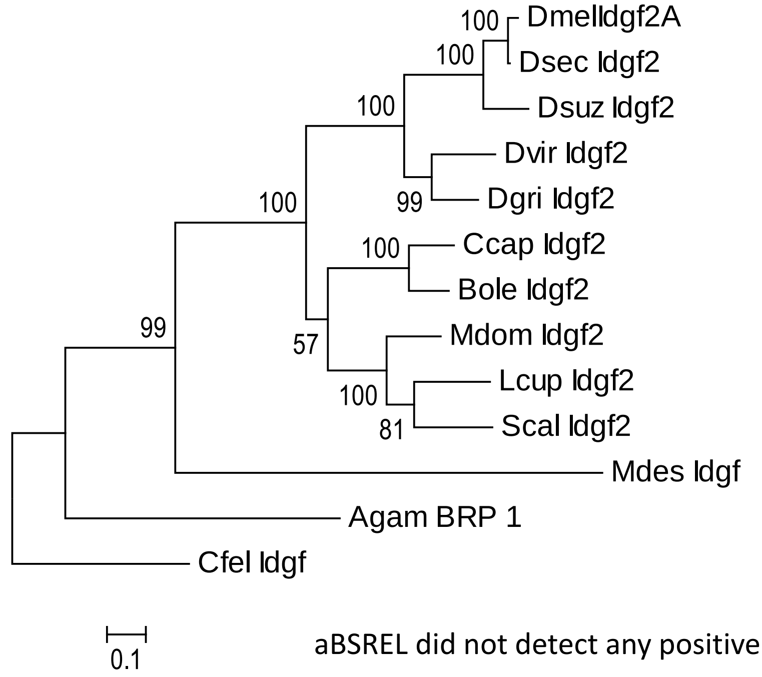

B aBSREL did not detect any positively selected nodes

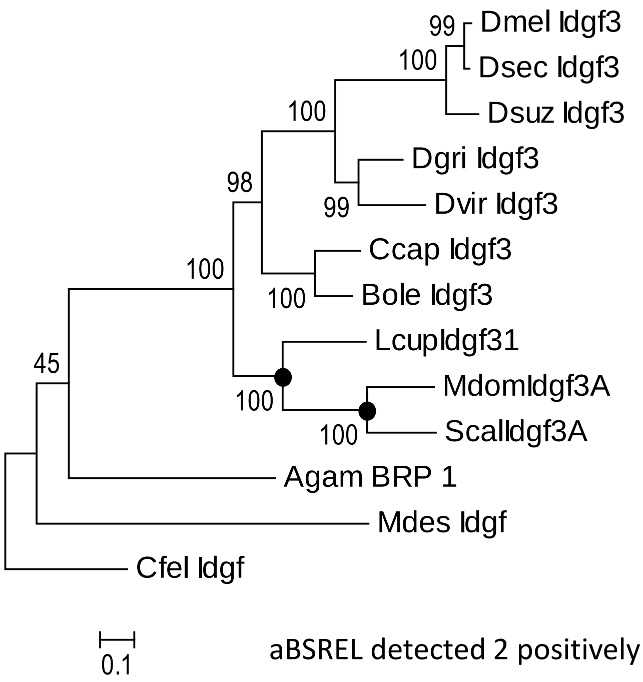

C aBSREL detected 2 positively selected nodes

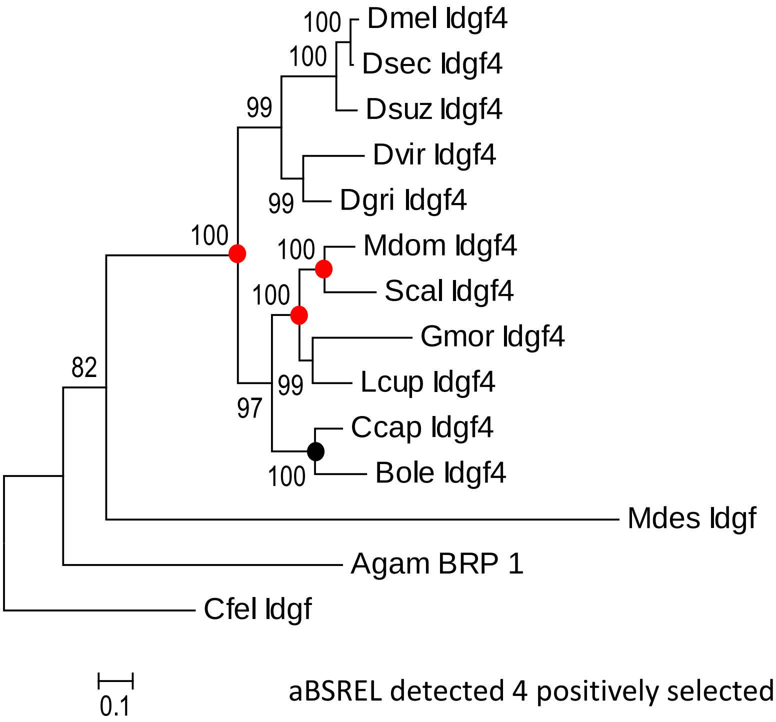

D aBSREL detected 4 positively selected nodes

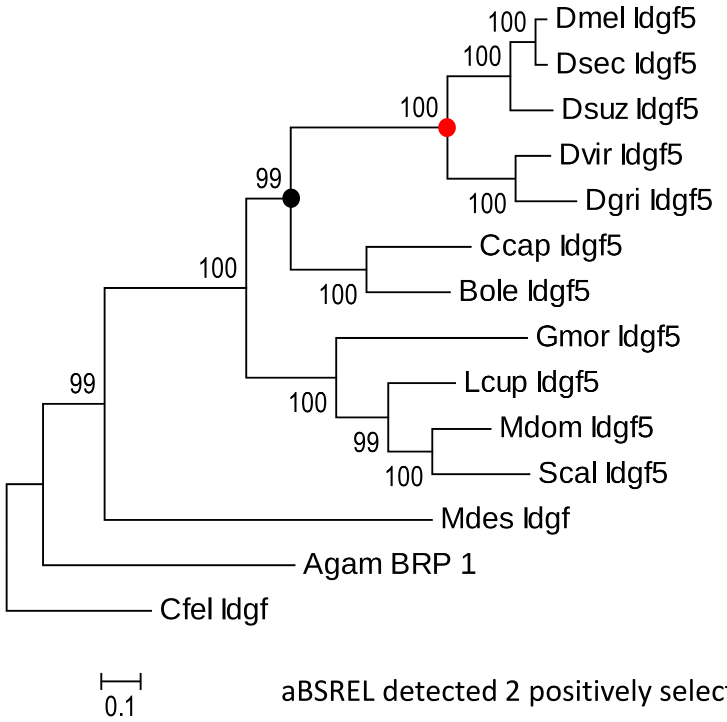

E

aBSREL detected 2 positively selected nodes

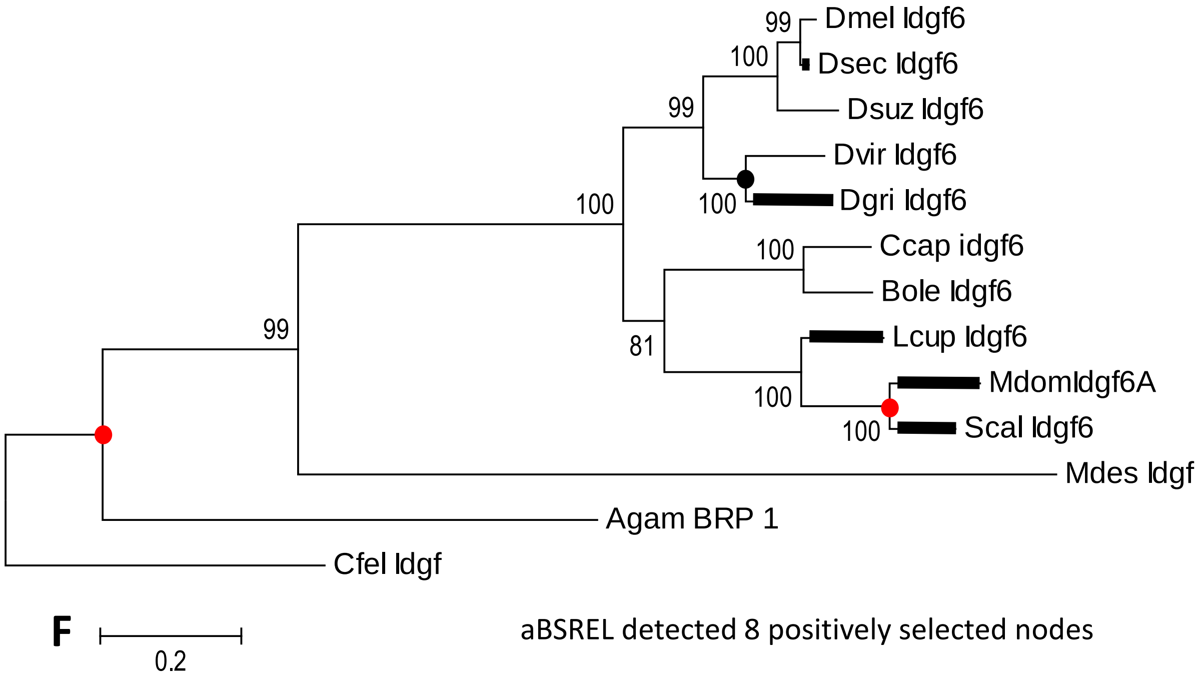

F

aBSREL detected 8 positively selected nodes

Figure S3G and H

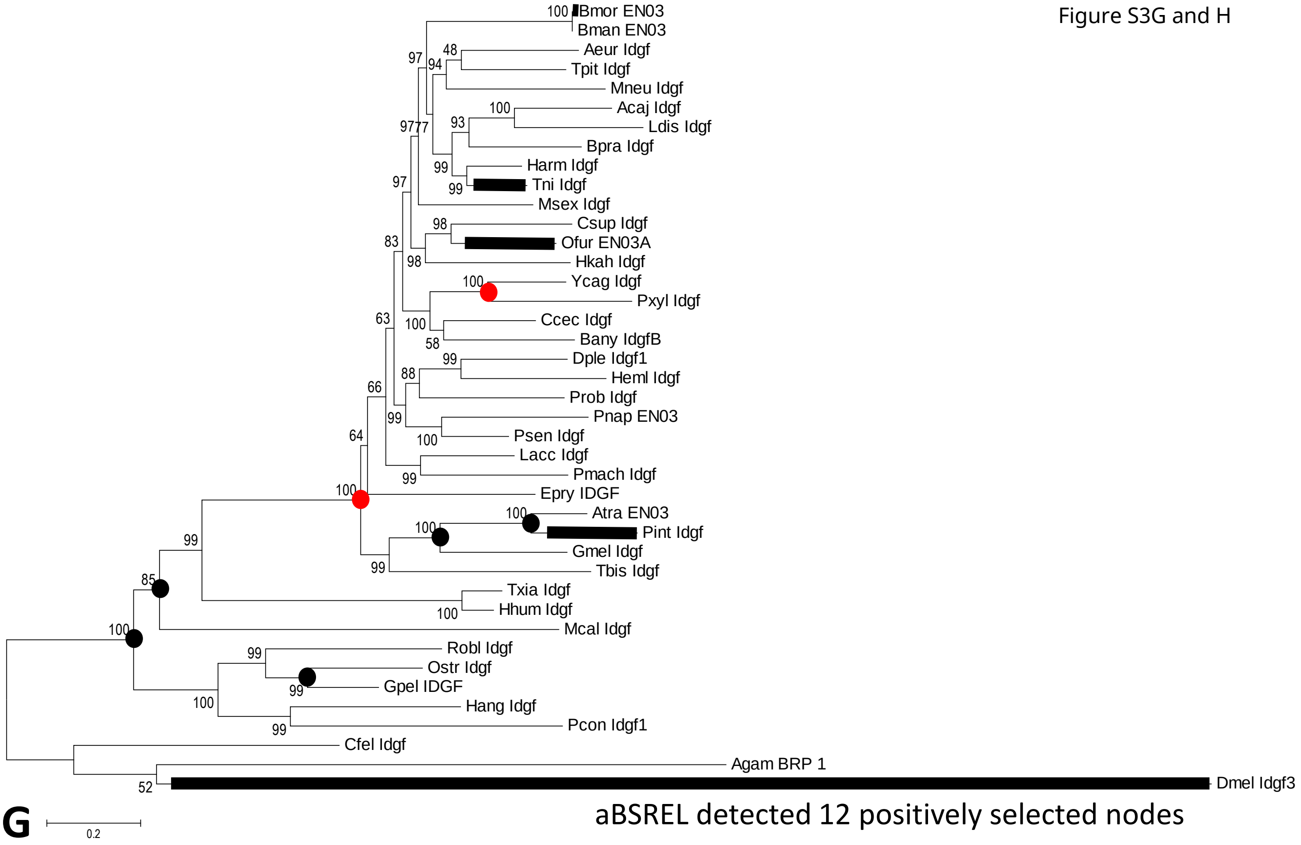

G

aBSREL detected 12 positively selected nodes

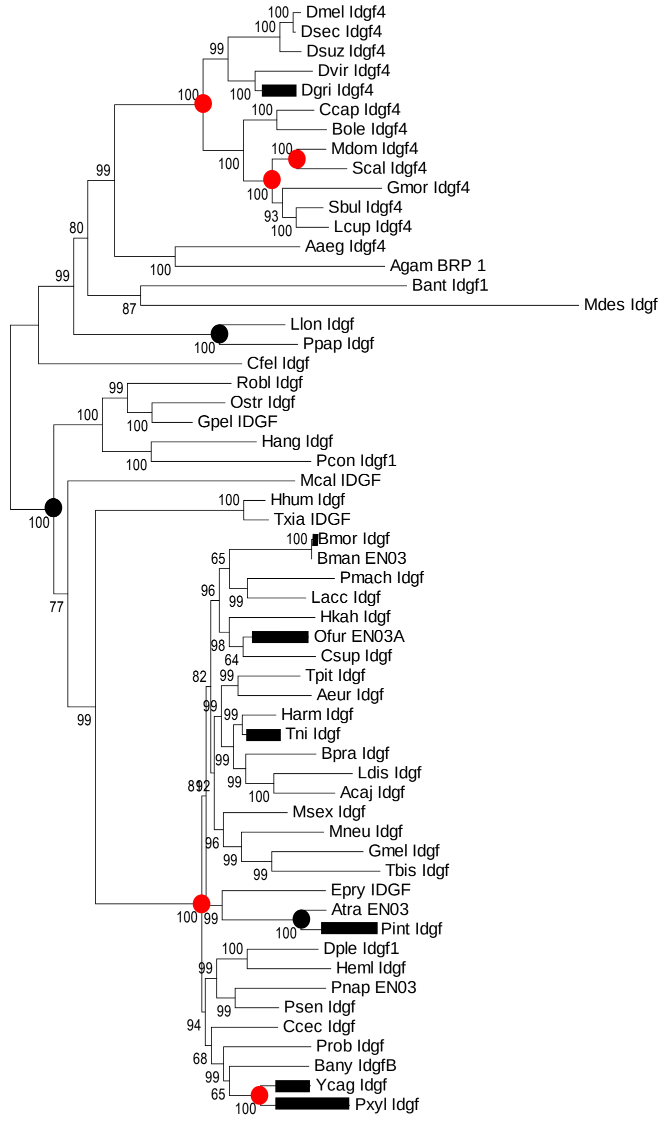

H

0.2

aBSREL detected 15 positively selected nodes

Figure S4

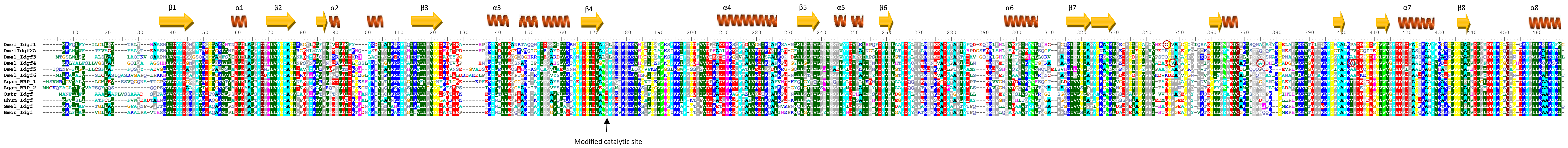
β1
α1
β2
α2
β3
α3
β4
α4
β5
α5
β6
α6
β7
α7
β8
α8
Dmel_Idgf1
DmelIdgf2A
Dmel_Idgf3
Dmel_Idgf4
Dmel_Idgf5
Dmel_Idgf6
Agam_BRP_1
Agam_BRP_2
Ostr_Idgf
Hhum_Idgf
Gmel_Idgf
Bmor_Idgf
Modified catalytic site

Table S1

Order	Family	Abbreviation	Species	Sequence ID	Database
Diptera	Drosophilidae	Dmel	Drosophila melanogaster	NM_057910, NM_057909, NM_165158, NM_001298139, NM_137477, NM_001299570	Genbank
		Dsec	Drosophila sechellia	XM_002038782, XM_002038781, XM_002038780, XM_002041783, XM_002034130, XM_002034398	Genbank
		Dsuz	Drosophila suzukii	XM_017090266, XM_017089326, XM_017089327, XM_017073697, XM_017081820, XM_017084939	Genbank
		Dvir	Drosophila virilis	XM_002052219, XM_002052220, XM_002052221, XM_002055522, XM_002048975, XM_002055522, XM_002050955	Genbank
		Dgri	Drosophila grimshawi	XM_001996410, XM_001996409, XM_001996408, XM_001997796, XM_001991865, XM_001995267, XM_001986951	Genbank
	Muscidae	Mdom	Musca domestica	XM_005179350, XM_005179351, XM_005179325, XM_005189010, XM_005188622, XM_005186242, XM_005181848	Genbank
		Scal	Stomoxys calcitrans	XM_013243787, XM_013243805, XM_013243807, XM_013251754, XM_013243813, XM_013253991, XM_013252596, XM_013262691	Genbank
	Sarcophagidae	Sbul	Sarcophaga bullata	TMW40086, TMW40679, TMW44785, TMW48511, TMW54608	Genbank
	Caliphoridae	Lcup	Lucilia cuprina	XM_023445243, XM_023445045, XM_023447952, XM_023440331, XM_023440337, XM_023440336, XM_023444827, XM_023447952	Genbank
	Glossinidae	Gmor	Glossina morsitans	DQ307194, DQ307195, DQ307196, DQ307197, DQ307193	Genbank
	Tephritidae	Ccap	Ceratitis capitata	XM_004521576, XM_004520878, XM_004518515, XM_004517420, XM_004518514, XM_004518516	Genbank
		Bole	Bactrocera oleae	XM_014247671, XM_014229710, XM_014243820, XM_014244863, XM_014247632	Genbank
	Cecidomyiidae	Mdes	Mayetiola destructor	Mdes001216	EnsemblM
	Chironomidae	Bant	Belgica antarctica	JU25_03746, JU25_04757	EnsemblM
	Culicidae	Aaeg	Aedes aegyptii	XM_001660695, XM_001660698	Genbank
		Agam	Anopheles gambiae	AY496420, AY496421	Genbank
	Psychodidae	Llon	Lutzomyia longipalpis	KY274439	Genbank
		Ppap	Phlebotomus papatasi	PPAI009732	Vector ba
Siphonaptera	Pulicidae	Cfel	Ctenocephalides felis	XM_026609733	Genbank
Trichoptera	Polycentropidae	Pcon	Plectrocnemia conspersa	MN520319, MN520320, MN520321	this study
	Hydropsychidae	Hang	Hydropsyche angustipennis	MN520322	this study
	Limnephilidae	Gpel	Glyphotaelius pellucidus	MN520337	this study
	Phryganeidae	Ostr	Oligotricha striata	MN520323	this study
	Rhyacophilidae	Robl	Rhyacophila obliterata	MN520324	this study
Lepidoptera	Micropterigidae	Mcal	Micropterix calthella	MN520338	this study
	Hepialidae	Hhum	Hepialus humuli	MN520325	this study
		Txia	Thitarodes xiaojinensis	MN520339	this study
	Tineidae	Tbis	Tineola bisseliella	MN520326	this study
	Psychidae	Ejap	Eumeta japonica	GBP97637	Genbank
		Epry	Eumata pryerii	MN520329	this study
	Yponomeutidae	Ycag	Yponomeuta cagnagella	MN520328	this study
	Plutellidae	Pxyl	Plutella xylostella	pacbiov1_core_32_85_1__cds__g12542	Lepbase
	Gracillariidae	Prob	Phyllonoricter roboris	MN520327	this study
	Pyralidae	Gmel	Galleria mellonella	MG846880	Genbank
		Pint	Plodia interpunctella	v1_core_32_85_1__cds__maker-scaffold10-augustus-gene-6.74-mRNA-1	Lepbase
		Atra	Amyelois transitella	XM_013333909	Genbank
	Crambidae	Ofur	Ostrinia furnacalis	XM_028300489	Genbank
		Csup	Chilo suppressalis	CSUOGS101503	Lepbase
	Nymphalidae	Dple	Danaus plexippus	v3_core_32_85_1_Idgf1	Lepbase
		Bany	Bicyclus anynana	v1x2_core_32_85_1__cds__BANY.1.2.t14035	Lepbase
		Hmel	Heliconius melpomene	hmel2_core_32_85_1__cds__HMEL012202g1	Lepbase
	Pieridae	Psen	Phoebis sennae	v1x1_core_32_85_1__cds__pse1789	Lepbase
		Pnap	Pieris napi	PIENAPT00000007661	Lepbase
	Papilionidae	Pmach	Papilio machaon	papma1_core_32_85_1__cds__KPJ09136	Lepbase
	Hesperiidae	Lacc	Lerema accius	v1x1_core_32_85_1__cds__lac161866	Lepbase
	Lycaenidae	Ccec	Calycopis cecrops	v1x1_core_32_85_1__cds__cce19999	Lepbase
	Lasiocampidae	Mneu	Malacosoma neustria	MN520334	this study
	Cosmopterigidae	Hkah	Hyposmocoma kahamanoa	XM_026463826	Genbank
	Bombycidae	Bmor	Bombyx mori	XM_021350318	Genbank
		Bman	Bombyx mandarina	XM_028176361	Genbank
	Sphingidae	Msex	Manduca sexta	GQ843826	Genbank
	Brahmaeidae	Aeur	Acanthobrahmaea europaea	MN520336	this study
	Lasiocampidae	Lque	Lasiocampa quercus	MN520335	this study
	Thaumetopoeidae	Tpit	Thaumatopoea pityocampa	MN520330	this study
	Lymantriidae	Ldis	Lymantria dispar	MN520333	this study
	Arctiidae	Acaj	Arctia caja	MN520331	this study
	Nolidae	Bpra	Bena prasinana	MN520332	this study
	Noctuidae	Tni	Trichoplusia ni	trichoplusia_ni_hi5v02_core_32_85_1__cds__evm	Lepbase
		Harm	Helicoverpa armigera	XM_021340699	Genbank
Coleoptera	Tenebrionidae	Tcas	Tribolium castaneum	NM_001044626	Genbank
	Scarabaeidae	Otau	Onthophagus taurus	XM_023052363	Genbank
	Buprestidae	Apla	Agrilus planipennis	XM_018473779	Genbank
	Curculionidae	Dpon	Dendroctonus ponderosae	XM_019911548	Genbank
Hymenoptera	Orussidae	Oabi	Orussus abietinus	XM_012432960	Genbank
	Braconidae	Hheb	Habrobracon hebetor	MG733033	Genbank
	Apidae	Bimp	Bombus impatiens	XM_003489324	Genbank
	Megachilidae	Mrot	Megachile rotundata	XM_012283507	Genbank
Thysanoptera	Tripidae	Focc	Frankliniella occidentalis	XM_026420798, XM_026436107	Genbank
Hemiptera	Pseudococcidae	Mhir	Maconellicoccus hirsutus	EF070598	Genbank
	Aphididae	Apis	Acyrthosiphon pisum	NM_001168671	Genbank
	Delphacidae	Nlug	Nilaparvata lugens	KM217119	Genbank
	Pentatomidae	Hhal	Halyomorpha halys	XM_024363492	Genbank
	Cicadellidae	Onig	Oncometopia nigricans	AY725777	Genbank
Orthoptera	Gryllidae	Gsig	Gryllodes sigillatus	KT355866	Genbank
Isoptera	Termopsidae	Znev	Zootermopsis nevadensis	XM_022084000	Genbank
	Kalotermitidae	Csec	Cryptotermes secundus	XM_023870763	Genbank

Name	B	LRT	Test p-value	Uncorrected p-value	ω distribution over sites
Node117	0.1379	29.9228	0	0	ω1 = 0.770 (84%)<br>ω2 = 226 (16%)
Node140	0.0375	45.4859	0	0	ω1 = 1.00 (91%)<br>ω2 = 208 (9.0%)
Node163	0.0696	76.0228	0	0	ω1 = 0.00 (79%)<br>ω2 = 30.0 (21%)
Node175	0.0382	43.5013	0	0	ω1 = 1.00 (94%)<br>ω2 = 95700000000 (6.4%)
Node183	0.074	73.8305	0	0	ω1 = 1.00 (88%)<br>ω2 = 1830 (12%)
Node225	0.0779	43.9947	0	0	ω1 = 0.0000000000577 (75%)<br>ω2 = 394 (25%)
Node25	0.0501	61.2561	0	0	ω1 = 1.00 (83%)<br>ω2 = 159 (17%)
Node35	0.0222	30.9761	0	0	ω1 = 0.809 (90%)<br>ω2 = 368 (9.5%)
Node40	0.0616	54.5886	0	0	ω1 = 1.00 (90%)<br>ω2 = 820 (9.6%)
Node186	0.0597	28.4148	0.0001	0	ω1 = 1.00 (7.3%)<br>ω2 = 1.00 (83%)<br>ω3 = 236 (9.5%)
Node38	0.0514	25.2506	0.0003	0	ω1 = 1.00 (89%)<br>ω2 = 663 (11%)
Node89	0.0551	24.129	0.0005	0	ω1 = 1.00 (92%)<br>ω2 = 613 (8.3%)
Node108	0.0786	23.1098	0.0009	0	ω1 = 0.00 (83%)<br>ω2 = 611 (17%)
Node151	0.0133	21.8992	0.0016	0	ω1 = 0.00 (97%)<br>ω2 = 2180 (3.2%)
Node32	0.0071	21.7488	0.0017	0	ω1 = 0.335 (97%)<br>ω2 = 273 (2.7%)
Node31	0.0113	21.504	0.0019	0	ω1 = 0.00 (91%)<br>ω2 = 400 (8.9%)
Node284	0.0324	20.3779	0.0034	0	ω1 = 0.975 (97%)<br>ω2 = 1160 (2.8%)
Node152	0.0493	17.8957	0.0118	0	ω1 = 0.210 (94%)<br>ω2 = 44.8 (6.1%)
Node37	0.0052	17.5	0.0143	0.0001	ω1 = 1.00 (95%)<br>ω2 = 1180 (5.1%)
DMEL_IDGF1	0.0282	17.0203	0.0182	0.0001	ω1 = 0.140 (98%)<br>ω2 = 5000 (1.5%)
Node64	0.0866	15.226	0.0447	0.0002	ω1 = 0.431 (87%)<br>ω2 = 116 (13%)

Table S2

B – optimized branch length, LRT – likelihood-ratio test, $\omega > 1$ – positive selection, $\omega < 1$ – negative selection, $\omega = 1$ – neutral evolution

Table S3A

Tree	Codon#	Position	Branch	Amount	Properties… (magnitude value, probability)			
Fig.S3D	7	Signal peptide	Idgf4	1	(6, .05) Polarity			
Fig.S3D	9	Signal peptide	Idgf4	3	(6, .001) Beta-structure tendencies	(7, .001) Polarity	(8, .01) Solvent accessible reduction ratio	
Fig.S3D	14	Signal peptide	Idgf4	1	(6, .001) Beta-structure tendencies			
Fig.S3D	18	Signal peptide	Idgf4	1	(6, .001) Beta-structure tendencies			
Fig.S3D	22	Signal peptide	Idgf4	1	(6, .001) Solvent accessible reduction ratio			
Fig.S3D	31	Signal peptide	Idgf4	1	(6, .001) Alpha-helical tendencies			
Fig.S3D	41	α helix 1	Idgf4	1	(6, .001) Alpha-helical tendencies			
Fig.S3D	103	α helix 2	Idgf4	1	(6, .001) Beta-structure tendencies			
Fig.S3D	110	α helix 2	Idgf4	1	(6, .001) Alpha-helical tendencies			
Fig.S3D	131	between β3-α3	Idgf4	1	(6, .01) Buriedness			
Fig.S3D	134	between β3-α3	Idgf4	4	(6, .001) Beta-structure tendencies	(8, .001) Bulkiness	(6, .001) Solvent accessible reduction ratio	(8, .001) Turn tendencies
Fig.S3D	153	α helix 3	Idgf4	1	(6, .01) Buriedness			
Fig.S3D	205	α helix 4	Idgf4	1	(7, .001) Polarity			
Fig.S3D	254	α helix 5	Idgf4	1	(6, .001) Beta-structure tendencies			
Fig.S3D	278	β sheet 6	Idgf4	4	(8, .05) Beta-structure tendencies	(7, .001) Buriedness	(8, .001) Polarity	(6, .001) Solvent accessible reduction ratio
Fig.S3D	299	α helix 6	Idgf4	1	(6, .001) Solvent accessible reduction ratio			
Fig.S3D	315	β sheet 7	Idgf4	4	(7, .001) Buriedness	(8, .001) Polarity	(8, .01) Solvent accessible reduction ratio	(8, .001) Turn tendencies
Fig.S3D	345	α7β7 DI*	Idgf4	2	(8, .001) Alpha-helical tendencies	(7, .001) Turn tendencies		
Fig.S3D	371	α7β7 DI*	Idgf4	2	(8, .001) Alpha-helical tendencies	(7, .001) Turn tendencies		
Fig.S3D	399	α7β7 DI*	Idgf4	2	(6, .001) Bulkiness	(7, .001) Turn tendencies		
Fig.S3D	400	α7β7 DI*	Idgf4	2	(8, .001) Alpha-helical tendencies	(7, .001) Turn tendencies		
Fig.S3D	459	α helix 8	Idgf4	1	(6, .001) Solvent accessible reduction ratio			

*α7β7 domain insertion

Table S3B

Tree	Codon#	Position	Branch	Amount	Properties… (magnitude value, probability)			
Fig.S3G	106	α helix 2	Lepidoptera	2	(6, .001) Polarity	(6, .001) Turn tendencies		
Fig.S3G	156	α helix 3	Lepidoptera	1	(8, .01) Alpha-helical tendencies			
Fig.S3G	196	loop between β4-α4**	Lepidoptera	1	(6, .001) Buriedness			
Fig.S3G	206	loop between β4-α4**	Lepidoptera	1	(6, .001) Turn tendencies			
Fig.S3G	228	between α4-β5	Lepidoptera	1	(7, .001) Polarity			
Fig.S3G	237	β sheet 5	Lepidoptera	2	(7, .001) Buriedness	(7, .001) Polarity		
Fig.S3G	240	β sheet 5	Lepidoptera	1	(6, .001) Beta-structure tendencies			
Fig.S3G	248	α helix 5	Lepidoptera	1	(6, .001) Alpha-helical tendencies			
Fig.S3G	260	between α5-β6	Lepidoptera	4	(6, .001) Buriedness	(8, .001) Polarity	(6, .001) Solvent accessible reduction ratio	(8, .001) Turn tendencies
Fig.S3G	289	between β6-α6	Lepidoptera	1	(6, .001) Alpha-helical tendencies			
Fig.S3G	292	between β6-α6	Lepidoptera	1	(8, .01) Alpha-helical tendencies			
Fig.S3G	296	between β6-α6	Lepidoptera	2	(7, .001) Buriedness	(7, .001) Polarity		
Fig.S3G	299	between β6-α6	Lepidoptera	4	(6, .001) Buriedness	(8, .001) Polarity	(6, .001) Solvent accessible reduction ratio	(8, .001) Turn tendencies
Fig.S3G	303	α helix 6	Lepidoptera	2	(6, .001) Alpha-helical tendencies	(6, .001) Turn tendencies		
Fig.S3G	472	between α7-β8	Lepidoptera	2	(6, .001) Alpha-helical tendencies	(6, .001) Turn tendencies		
Fig.S3G	479	β sheet 8	Lepidoptera	2	(6, .001) Alpha-helical tendencies	(6, .001) Turn tendencies		

**characteristic fo Idgfs

Table S3C

Tree	Codon#	Position	Branch	Amount	Properties… (magnitude value, probability)	
Fig.S3G	13	Signal peptide	Yponomutoidea	1	(6, .001) Solvent accessible reduction ratio	
Fig.S3G	29	before β1	Yponomutoidea	1	(6, .001) Beta-structure tendencies	
Fig.S3G	185	loop between β4-α4**	Yponomutoidea	1	(6, .01) Bulkiness	
Fig.S3G	295	between β6-α6	Yponomutoidea	1	(6, .001) Polarity	
Fig.S3G	326	β sheet 7	Yponomutoidea	1	(8, .01) Alpha-helical tendencies	
Fig.S3G	338	β sheet 7	Yponomutoidea	2	(6, .001) Alpha-helical tendencies	(6, .001) Turn tendencies
Fig.S3G	490	between β8-α8	Yponomutoidea	1	(8, .01) Alpha-helical tendencies	

**characteristic fo Idgfs

Purifying selection was confirmed by SELECTON, FEL and FUBAR analysis in the following proportion of positions:

	Number of negatively selected positions/length of alignment	ratio
Schizophora Idgf1	353/470	0,75
Schizophora Idgf2	355/469	0,75
Schizophora Idgf3	347/469	0,74
Schizophora Idgf4	365/466	0,78
Schizophora Idgf5	321/478	0,67
Schizophora Idgf6	314/497	0,63
Lepidoptera	413/508	0,81

Alignment Position	Refseq Hhum	Selecton Ka/Ks	Refseq Position	Ka/Ks	FEL ω>1 ω<1	p-value	FUBAR neg/purif P[α>β]	pos/div P[α<β]
1	M	0.0055	1	0.0055	NaN	1	0.97	0.018
2	W	0.11	2	0.11	0.225	0.242	0.938	0.049
3	V	0.04	3	0.04	0.115	0.004	1	0
5	S	0.4	5	0.4	0.175	0.005	0.983	0
6	L	0.14	6	0.14	0.007	0	1	0
7	I	0.25	7	0.25	0.183	0.001	1	0
9	-	0.2			0.05	0.145	0.929	0.057
10	-	0.03			0	0.147	0.93	0.056
11	-	0.022			0	0.009	0.995	0.004
12	A	0.32	8	0.32	0.234	0.012	0.982	0
13	T	0.27	9	0.27	0.146	0.001	0.999	0
14	F	0.2	10	0.2	0.687	0.404	0.96	0.012
15	C	0.13	11	0.13	0.199	0	1	0
16	L	0.38	12	0.38	0.571	0.2	0.981	0.005
17	L	0.25	13	0.25	0.301	0.006	0.999	0
18	F	0.07	14	0.07	0.164	0	1	0
19	V	0.39	15	0.39	0.561	0.26	0.991	0.001
20	W	0.15	16	0.15	0.111	0	1	0
21	T	0.2	17	0.2	0.222	0.002	0.996	0
22	E	0.088	18	0.088	0.374	0.036	0.964	0.016
24	-	0.38			0.246	0.006	0.998	0
29	D	0.35	20	0.35	0.114	0	1	0
30	T	0.4	21	0.4	0.357	0.083	0.947	0
32	H	0.36	23	0.36	0.492	0.313	0.926	0.014
33	N	0.23	24	0.23	0.409	0.075	0.931	0.007
34	K	0.043	25	0.043	0.061	0	1	0
35	V	0.017	26	0.017	0.011	0	1	0
37	C	0.0036	28	0.0036	0	0	1	0
38	Y	0.027	29	0.027	0.068	0	0.997	0.001
39	Y	0.0033	30	0.0033	0	0	1	0
40	D	0.019	31	0.019	0.035	0	1	0
42	R	0.075	33	0.075	0.226	0.024	0.986	0.009
43	S	0.0098	34	0.0098	0.017	0	1	0
44	F	0.078	35	0.078	0.136	0.001	0.983	0.009
46	R	0.0021	37	0.0021	0	0	1	0
47	E	0.01	38	0.01	0.029	0	1	0
48	-	0.035			0	0.003	0.997	0.002
49	-	0.023			0	0.001	0.999	0.001
51	-	0.032			0	0.044	0.98	0.015
56	Q	0.029	40	0.029	0.174	0.016	0.992	0.005
57	G	0.022	41	0.022	0.046	0	1	0
58	K	0.023	42	0.023	0.029	0	1	0
59	M	0.026	43	0.026	0	0.009	0.998	0.001
60	I	0.064	44	0.064	0.237	0.014	0.974	0.014
61	L	0.052	45	0.052	0.031	0	1	0
62	A	0.23	46	0.23	0.604	0.292	0.968	0.011
63	D	0.0023	47	0.0023	0	0	1	0

Table S5

64 L	0.039	48	0.039	0.061	*0*	*1*	0
65 E	0.08	49	0.08	0.022	*0*	*1*	0
66 P	0.034	50	0.034	0.034	*0*	*1*	0
67 A	0.0018	51	0.0018	0	*0*	*1*	0
68 L	0.016	52	0.016	0.01	*0*	*1*	0
69 T	0.081	53	0.081	0.08	*0*	*1*	0
70 F	0.063	54	0.063	0.229	*0.077*	*0.909*	0.048
71 C	0.0035	55	0.0035	0	*0.001*	*0.998*	0
72 T	0.03	56	0.03	0.072	*0*	*1*	0
73 H	0.017	57	0.017	0.001	*0*	*1*	0
74 L	0.028	58	0.028	0.034	*0*	*1*	0
75 V	0.075	59	0.075	0.085	*0*	*1*	0
76 Y	0.0033	60	0.0033	0	*0*	*1*	0
77 G	0.0036	61	0.0036	0	*0*	*1*	0
78 Y	0.016	62	0.016	0.029	*0*	*0.999*	0
79 A	0.0017	63	0.0017	0	*0*	*1*	0
80 G	0.027	64	0.027	0.017	*0*	*1*	0
81 I	0.08	65	0.08	0.161	*0*	*0.999*	0.001
82 Q	0.093	66	0.093	0.009	*0*	*1*	0
83 P	0.038	67	0.038	0.079	*0*	*1*	0
84 D	0.047	68	0.047	0.11	*0.003*	*0.968*	0.02
85 T	0.018	69	0.018	0.035	*0*	*1*	0
86 Y	0.09	70	0.09	0.144	*0.002*	*0.972*	0.016
87 R	0.032	71	0.032	0.014	*0*	*1*	0
89 V	0.086	73	0.086	0.195	*0*	*1*	0
90 P	0.11	74	0.11	0.101	*0*	*1*	0
91 L	0.027	75	0.027	0.02	*0*	*1*	0
92 N	0.0016	76	0.0016	0	*0*	*1*	0
93 E	0.074	77	0.074	0.07	*0*	*1*	0
94 N	0.024	78	0.024	0.05	*0*	*1*	0
95 L	0.0036	79	0.0036	0	*0*	*1*	0
96 D	0.011	80	0.011	0.007	*0*	*1*	0
97 T	0.16	81	0.16	0.2	*0*	*1*	0
98 D	0.03	82	0.03	0.046	*0*	*1*	0
99 K	0.16	83	0.16	0.041	*0*	*1*	0
100 G	0.18	84	0.18	0.244	*0.002*	*0.999*	0
101 H	0.019	85	0.019	0.009	*0*	*1*	0
102 A	0.09	86	0.09	0.361	*0.034*	*0.983*	0.007
103 L	0.024	87	0.024	0.042	*0*	*1*	0
104 Y	0.051	88	0.051	0.07	*0*	*0.999*	0
105 R	0.023	89	0.023	0.013	*0*	*1*	0
106 N	0.18	90	0.18	0.268	*0.005*	*0.998*	0
107 V	0.038	91	0.038	0.092	*0*	*1*	0
108 A	0.012	92	0.012	0.047	*0*	*1*	0
109 A	0.068	93	0.068	0.121	*0.001*	*0.987*	0.007
111 N	0.029	95	0.029	0.053	*0*	*1*	0
112 K	0.18	96	0.18	0.459	*0.077*	*0.988*	0.002
113 K	0.012	97	0.012	0.002	*0*	*1*	0
114 Y	0.067	98	0.067	0.037	*0*	*1*	0
115 P	0.014	99	0.014	0.012	*0*	*1*	0

116 G	0.16	100	0.16	0.179	*0*	*1*	0
117 L	0.041	101	0.041	0.036	*0*	*1*	0
118 K	0.022	102	0.022	0.001	*0*	*1*	0
119 V	0.032	103	0.032	0.014	*0*	*1*	0
120 L	0.071	104	0.071	0.047	*0*	*1*	0
121 L	0.0036	105	0.0036	0	*0*	*1*	0
122 S	0.026	106	0.026	0.03	*0*	*1*	0
123 V	0.023	107	0.023	0.041	*0*	*1*	0
124 G	0.0036	108	0.0036	0	*0*	*1*	0
125 G	0.033	109	0.033	0.046	*0*	*1*	0
126 G	0.043	110	0.043	0.043	*0*	*1*	0
127 A	0.084	111	0.084	0.165	*0*	*1*	0
128 D	0.0025	112	0.0025	0	*0*	*1*	0
130 K	0.22	113	0.22	0.392	*0.039*	*0.952*	0.011
131 E	0.082	114	0.082	0.005	*0*	*1*	0
132 E	0.23	115	0.23	0.045	*0*	*1*	0
133 K	0.49	116	0.49	0.336	*0.02*	*0.991*	0
134 E	0.18	117	0.18	0.17	*0.001*	*1*	0
137 K	0.012	118	0.012	0.005	*0*	*1*	0
138 Y	0.0033	119	0.0033	0	*0*	*1*	0
139 N	0.032	120	0.032	0.024	*0*	*1*	0
140 L	0.089	121	0.089	0.123	*0*	*1*	0
141 L	0.038	122	0.038	0.046	*0*	*1*	0
142 L	0.015	123	0.015	0.011	*0*	*1*	0
143 E	0.0023	124	0.0023	0	*0*	*1*	0
144 S	0.023	125	0.023	0.03	*0*	*1*	0
145 P	0.13	126	0.13	0.112	*0*	*1*	0
147 E	0.054	127	0.054	0.2	*0.014*	*0.991*	0.006
148 A	0.061	128	0.061	0.091	*0*	*1*	0
149 R	0.033	129	0.033	0.064	*0*	*1*	0
150 T	0.037	130	0.037	0.06	*0*	*1*	0
151 K	0.062	131	0.062	0.009	*0*	*1*	0
152 F	0.027	132	0.027	0.007	*0*	*1*	0
153 I	0.029	133	0.029	0.032	*0*	*1*	0
154 N	0.031	134	0.031	0.108	*0.002*	*0.97*	0.017
155 S	0.013	135	0.013	0.052	*0*	*1*	0
156 A	0.075	136	0.075	0.126	*0*	*1*	0
157 V	0.083	137	0.083	0.062	*0*	*1*	0
158 Q	0.088	138	0.088	0.01	*0*	*1*	0
159 L	0.026	139	0.026	0.017	*0*	*1*	0
160 T	0.057	140	0.057	0.091	*0*	*1*	0
161 K	0.083	141	0.083	0.042	*0*	*1*	0
162 Q	0.038	142	0.038	0.011	*0*	*1*	0
163 Y	0.077	143	0.077	0.007	*0*	*1*	0
164 G	0.088	144	0.088	0.094	*0*	*1*	0
165 F	0.0034	145	0.0034	0	*0*	*1*	0
166 D	0.011	146	0.011	0.003	*0*	*1*	0
167 G	0.0032	147	0.0032	0	*0*	*1*	0
168 L	0.063	148	0.063	0.039	*0*	*1*	0
169 D	0.011	149	0.011	0.006	*0*	*1*	0

Type your text

170 I	0.054	150	0.054	0.022	*0*	*1*	0
171 A	0.014	151	0.014	0.034	*0*	*1*	0
172 W	0.035	152	0.035	0	*0.001*	*1*	0
173 E	0.034	153	0.034	0.031	*0*	*1*	0
174 F	0.038	154	0.038	0.141	*0.013*	*0.978*	0.009
175 P	0.12	155	0.12	0.096	*0*	*1*	0
176 E	0.18	156	0.18	0.153	*0*	*1*	0
177 N	0.17	157	0.17	0.34	*0.016*	*0.98*	0.006
178 K	0.0025	158	0.0025	0	*0*	*1*	0
179 P	0.0031	159	0.0031	0	*0*	*1*	0
180 K	0.011	160	0.011	0.022	*0*	*1*	0
181 K	0.021	161	0.021	0.03	*0*	*1*	0
182 I	0.046	162	0.046	0.168	*0.002*	*0.987*	0.007
183 R	0.012	163	0.012	0.023	*0*	*1*	0
184 G	0.038	164	0.038	0.082	*0*	*1*	0
185 A	0.17	165	0.17	0.383	*0.03*	*0.974*	0.009
186 I	0.38	166	0.38	0.016	*0*	*1*	0
187 G	0.032	167	0.032	0.076	*0*	*1*	0
188 S	0.07	168	0.07	0.017	*0*	*1*	0
189 L	0.2	169	0.2	0.37	*0.066*	*0.925*	0.04
190 W	0.0073	170	0.0073	NaN	1	*0.984*	0.003
191 H	0.01	171	0.01	0.008	*0*	*1*	0
192 G	0.075	172	0.075	0.058	*0*	*1*	0
193 F	0.084	173	0.084	0.258	*0.026*	*0.913*	0.056
194 K	0.0025	174	0.0025	0	*0*	*1*	0
195 K	0.013	175	0.013	0.016	*0*	*1*	0
196 I	0.078	176	0.078	0.101	*0*	*1*	0
197 F	0.0033	177	0.0033	0	*0*	*1*	0
200 K	0.076	178	0.076	0.192	*0.001*	*0.995*	0.002
201 T	0.073	179	0.073	0.07	*0*	*1*	0
202 T	0.033	180	0.033	0.033	*0*	*1*	0
203 P	0.076	181	0.076	0.122	*0*	*1*	0
204 V	0.026	182	0.026	0.019	*0*	*1*	0
205 D	0.0024	183	0.0024	0	*0*	*1*	0
206 E	0.17	184	0.17	0.25	*0.042*	*0.904*	0.063
207 N	0.032	185	0.032	0.032	*0*	*1*	0
208 A	0.024	186	0.024	0.018	*0*	*1*	0
209 E	0.17	187	0.17	0.338	*0.017*	*0.983*	0.006
210 Q	0.03	188	0.03	0.027	*0*	*1*	0
211 H	0.002	189	0.002	0	*0*	*1*	0
212 R	0.042	190	0.042	0.035	*0*	*1*	0
213 E	0.075	191	0.075	0.153	*0.003*	*0.998*	0.001
214 G	0.054	192	0.054	0.052	*0*	*1*	0
215 Y	0.059	193	0.059	0.03	*0*	*1*	0
216 T	0.019	194	0.019	0.033	*0*	*1*	0
217 A	0.031	195	0.031	0.025	*0*	*1*	0
218 F	0.027	196	0.027	0.018	*0*	*1*	0
219 I	0.021	197	0.021	0.03	*0*	*1*	0
220 R	0.033	198	0.033	0.044	*0*	*1*	0
221 E	0.02	199	0.02	0.024	*0*	*1*	0

222 L	0.15	200	0.15	0.306	*0.049*	*0.966*	0.019
223 K	0.034	201	0.034	0.023	*0*	*1*	0
224 Q	0.074	202	0.074	0.014	*0*	*1*	0
225 A	0.0072	203	0.0072	0.011	*0*	*1*	0
226 V	0.091	204	0.091	0.034	*0*	*1*	0
227 R	0.11	205	0.11	0.083	*0*	*1*	0
228 P	0.086	206	0.086	0.093	*0*	*1*	0
233 E	0.038	207	0.038	0.03	*0*	*1*	0
234 -	0.024			0.015	*0*	*1*	0
235 K	0.067	208	0.067	0.09	*0*	*0.998*	0.001
236 L	0.14	209	0.14	0	*0.001*	*0.999*	0.001
237 I	0.09	210	0.09	0.069	*0*	*1*	0
238 L	0.028	211	0.028	0.004	*0*	*1*	0
239 T	0.11	212	0.11	0.207	*0*	*1*	0
241 T	0.046	214	0.046	0.091	*0*	*1*	0
242 V	0.053	215	0.053	0.056	*0*	*1*	0
243 L	0.033	216	0.033	0.019	*0*	*1*	0
244 P	0.0028	217	0.0028	0	*0*	*1*	0
245 N	0.021	218	0.021	0.018	*0*	*1*	0
246 V	0.0086	219	0.0086	0.024	*0*	*1*	0
247 N	0.0017	220	0.0017	0	*0*	*1*	0
249 T	0.13	222	0.13	0.262	*0.007*	*1*	0
250 I	0.15	223	0.15	0.127	*0*	*1*	0
251 Y	0.037	224	0.037	0.184	*0.057*	*0.916*	0.045
253 D	0.0024	226	0.0024	0	*0*	*1*	0
254 V	0.034	227	0.034	0.078	*0*	*1*	0
255 S	0.033	228	0.033	0.02	*0*	*1*	0
256 A	0.083	229	0.083	0.255	*0.004*	*0.996*	0.001
257 I	0.02	230	0.02	0.042	*0*	*1*	0
258 I	0.037	231	0.037	0.057	*0*	*1*	0
259 N	0.025	232	0.025	0.028	*0*	*1*	0
260 H	0.14	233	0.14	0.1	*0*	*1*	0
261 L	0.045	234	0.045	0.05	*0*	*1*	0
262 D	0.0023	235	0.0023	0	*0*	*1*	0
263 F	0.2	236	0.2	0.132	*0*	*0.999*	0
264 V	0.033	237	0.033	0.052	*0*	*1*	0
265 N	0.036	238	0.036	0.081	*0*	*0.997*	0.001
266 L	0.16	239	0.16	0.014	*0*	*1*	0
267 E	0.39	240	0.39	0.317	*0.08*	*0.938*	0.009
268 G	0.059	241	0.059	0.057	*0*	*1*	0
269 F	0.14	242	0.14	0.01	*0*	*1*	0
270 D	0.01	243	0.01	0.011	*0*	*1*	0
271 F	0.15	244	0.15	0.098	*0*	*0.993*	0.004
273 T	0.0072	246	0.0072	0.007	*0*	*1*	0
274 P	0.05	247	0.05	0.037	*0*	*1*	0
275 E	0.11	248	0.11	0.008	*0*	*1*	0
276 R	0.0025	249	0.0025	0	*0*	*1*	0
277 N	0.0016	250	0.0016	0	*0*	*1*	0
278 P	0.039	251	0.039	0.055	*0*	*1*	0
279 S	0.036	252	0.036	0.015	*0*	*1*	0

280 E	0.01	253	0.01	0.003	0	1	0
281 A	0.014	254	0.014	0.032	0	1	0
282 D	0.0023	255	0.0023	0	0	1	0
283 Y	0.026	256	0.026	0.023	0	1	0
284 T	0.085	257	0.085	0.208	0	1	0
285 A	0.13	258	0.13	0.197	0.001	1	0
286 P	0.003	259	0.003	0	0	1	0
287 L	0.14	260	0.14	0.048	0	1	0
288 Y	0.026	261	0.026	0.041	0	1	0
289 E	0.093	262	0.093	0.045	0	1	0
290 A	0.063	263	0.063	0.02	0	1	0
291 Y	0.083	264	0.083	0.09	0	1	0
292 E	0.062	265	0.062	0.235	0.019	0.896	0.068
293 R	0.011	266	0.011	0.01	0	1	0
294 N	0.03	267	0.03	0.037	0	1	0
295 P	0.17	268	0.17	0.266	0.005	0.998	0.001
296 G	0.16	269	0.16	0.171	0	1	0
299 N	0.28	270	0.28	0.042	0	1	0
300 N	0.0017	271	0.0017	0	0	1	0
301 I	0.11	272	0.11	0.17	0	1	0
302 N	0.067	273	0.067	0.025	0	1	0
303 H	0.12	274	0.12	0.263	0.002	0.997	0.001
304 L	0.1	275	0.1	0.208	0.001	1	0
305 T	0.056	276	0.056	0.143	0	1	0
306 Q	0.38	277	0.38	0.287	0.007	0.936	0
307 Y	0.072	278	0.072	0.204	0.027	0.912	0.059
308 W	0.057	279	0.057	0.005	0.015	0.991	0.007
309 L	0.18	280	0.18	0.058	0	1	0
310 T	0.19	281	0.19	0.063	0	1	0
311 N	0.19	282	0.19	0.184	0.001	0.982	0
312 G	0.11	283	0.11	0.158	0	0.999	0
313 A	0.054	284	0.054	0.011	0	1	0
314 P	0.015	285	0.015	0.002	0	1	0
315 G	0.31	286	0.31	0.161	0.001	0.983	0
316 S	0.31	287	0.31	0.233	0.015	0.964	0
317 K	0.0025	288	0.0025	0	0	1	0
318 I	0.092	289	0.092	0.004	0	1	0
319 V	0.074	290	0.074	0.114	0	1	0
320 V	0.19	291	0.19	0.12	0	1	0
321 G	0.07	292	0.07	0.117	0	0.999	0
322 I	0.068	293	0.068	0.152	0.002	0.987	0.007
323 A	0.079	294	0.079	0.119	0	1	0
324 T	0.035	295	0.035	0.061	0	1	0
325 F	0.2	296	0.2	0.055	0	1	0
326 G	0.14	297	0.14	0.171	0	1	0
327 R	0.0027	298	0.0027	0	0	1	0
328 T	0.042	299	0.042	0.065	0	1	0
329 W	0.0073	300	0.0073	NaN	1	0.984	0.003
330 K	0.032	301	0.032	0.002	0	1	0
331 M	0.076	302	0.076	0.088	0	1	0

332 T	0.061	303	0.061	0.067	*0*	*1*	0
333 S	0.17	304	0.17	0.322	*0.008*	*0.999*	0
334 S	0.082	305	0.082	0.126	*0.003*	*0.959*	0.026
335 S	0.038	306	0.038	0.018	*0*	*1*	0
336 N	0.068	307	0.068	0.041	*0*	*1*	0
337 I	0.027	308	0.027	0.069	*0*	*1*	0
338 A	0.076	309	0.076	0.25	*0.002*	*0.99*	0.004
339 G	0.004	310	0.004	0	*0*	*1*	0
340 V	0.0091	311	0.0091	0.009	*0*	*1*	0
341 P	0.0032	312	0.0032	0	*0*	*1*	0
342 P	0.034	313	0.034	0.035	*0*	*1*	0
343 L	0.23	314	0.23	0.507	0.113	*0.96*	0.013
344 E	0.083	315	0.083	0.075	*0*	*1*	0
346 M	0.21	317	0.21	0	0.399	*0.916*	0.071
347 D	0.068	318	0.068	0.038	*0*	*1*	0
348 G	0.017	319	0.017	0.011	*0*	*1*	0
349 P	0.13	320	0.13	0.212	*0*	*0.999*	0
350 G	0.031	321	0.031	0.027	*0*	*1*	0
351 E	0.037	322	0.037	0.148	*0.003*	*0.999*	0.001
352 E	0.029	323	0.029	0.092	*0*	*0.999*	0
353 G	0.0036	324	0.0036	0	*0*	*1*	0
354 P	0.0033	325	0.0033	0	*0*	*1*	0
355 Y	0.035	326	0.035	0.191	*0.055*	0.9	0.059
356 S	0.025	327	0.025	0.044	*0*	*1*	0
357 K	0.021	328	0.021	0.003	*0*	*1*	0
358 T	0.19	329	0.19	0.298	*0.004*	*0.999*	0
359 P	0.086	330	0.086	0.08	*0*	*1*	0
360 G	0.015	331	0.015	0.019	*0*	*1*	0
361 L	0.19	332	0.19	0.065	*0*	*1*	0
362 L	0.017	333	0.017	0.02	*0*	*1*	0
363 S	0.0055	334	0.0055	0.007	*0*	*1*	0
364 Y	0.041	335	0.041	0.044	*0*	*1*	0
365 P	0.042	336	0.042	0.032	*0*	*1*	0
366 E	0.0024	337	0.0024	0	*0*	*1*	0
367 V	0.043	338	0.043	0.03	*0*	*1*	0
368 C	0.0036	339	0.0036	0	*0*	*1*	0
369 A	0.078	340	0.078	0.188	*0*	*1*	0
370 K	0.013	341	0.013	0.012	*0*	*1*	0
371 L	0.015	342	0.015	0.013	*0*	*1*	0
373 N	0.02	344	0.02	0.02	*0*	*1*	0
374 P	0.0029	345	0.0029	0	*0*	*1*	0
375 D	0.083	346	0.083	0.088	*0*	*0.998*	0.001
376 N	0.029	347	0.029	0.029	*0*	*1*	0
377 Q	0.084	348	0.084	0.1	*0*	*1*	0
378 K	0.046	349	0.046	0.032	*0*	*1*	0
381 G	0.0038	350	0.0038	0	*0*	*1*	0
382 L	0.076	351	0.076	0.04	*0*	*1*	0
383 L	0.17	352	0.17	0.071	*0*	*1*	0
384 P	0.087	353	0.087	0.125	*0*	*1*	0
385 H	0.082	354	0.082	0.054	*0*	*1*	0

386 L	0.052	355	0.052	0.024	*0*	*1*	0
387 R	0.0088	356	0.0088	0.013	*0*	*1*	0
388 K	0.02	357	0.02	0.015	*0*	*1*	0
389 V	0.021	358	0.021	0.045	*0*	*1*	0
390 P	0.07	359	0.07	0.155	*0*	*1*	0
391 D	0.0024	360	0.0024	0	*0*	*1*	0
392 P	0.0029	361	0.0029	0	*0*	*1*	0
393 S	0.014	362	0.014	0.173	*0.049*	*0.928*	0.034
394 K	0.029	363	0.029	0.01	*0*	*1*	0
395 R	0.0031	364	0.0031	0	*0*	*1*	0
396 F	0.044	365	0.044	0.014	*0*	*1*	0
433 -	0.067			0	0.115	*0.959*	0.033
434 -	0.052			0	*0.084*	*0.972*	0.023
438 G	0.0041	366	0.0041	0	*0*	*1*	0
439 T	0.015	367	0.015	0.03	*0*	*1*	0
440 Y	0.0033	368	0.0033	0	*0*	*1*	0
441 A	0.0017	369	0.0017	0	*0*	*1*	0
442 F	0.026	370	0.026	0.15	*0.082*	*0.921*	0.026
443 R	0.013	371	0.013	0.034	*0*	*1*	0
444 V	0.18	372	0.18	0.108	*0*	*1*	0
445 P	0.048	373	0.048	0.096	*0*	*1*	0
446 D	0.011	374	0.011	0.008	*0*	*1*	0
447 D	0.064	375	0.064	0.121	*0.002*	*0.961*	0.024
448 N	0.092	376	0.092	0.101	*0*	*0.998*	0.001
449 D	0.096	377	0.096	0.091	*0*	*1*	0
450 E	0.075	378	0.075	0.029	*0*	*1*	0
451 G	0.082	379	0.082	0.252	*0.003*	*0.972*	0.011
452 G	0.0034	380	0.0034	0	*0*	*1*	0
453 L	0.19	381	0.19	0.085	*0*	*1*	0
454 W	0.0073	382	0.0073	NaN	1	*0.984*	0.003
455 V	0.041	383	0.041	0.08	*0*	*1*	0
456 G	0.19	384	0.19	0.411	*0.091*	*0.956*	0.013
457 Y	0.0032	385	0.0032	0	*0*	*1*	0
458 E	0.01	386	0.01	0.001	*0*	*1*	0
459 D	0.0024	387	0.0024	0	*0*	*1*	0
460 P	0.048	388	0.048	0.027	*0*	*1*	0
461 D	0.036	389	0.036	0.051	*0*	*1*	0
462 S	0.028	390	0.028	0.044	*0*	*1*	0
463 A	0.0079	391	0.0079	0.031	*0*	*1*	0
464 G	0.086	392	0.086	0.188	*0.001*	*0.999*	0
465 D	0.04	393	0.04	0.062	*0*	*1*	0
466 K	0.02	394	0.02	0.007	*0*	*1*	0
467 A	0.014	395	0.014	0.027	*0*	*1*	0
469 Y	0.038	397	0.038	0.011	*0*	*1*	0
470 V	0.083	398	0.083	0.151	*0*	*1*	0
471 R	0.043	399	0.043	0.045	*0*	*1*	0
472 A	0.064	400	0.064	0.162	*0*	*1*	0
473 K	0.036	401	0.036	0.087	*0.001*	*1*	0
474 G	0.15	402	0.15	0.154	*0*	*1*	0
475 L	0.0032	403	0.0032	0	*0*	*1*	0

476 G	0.014	404	0.014	0.011	*0*	*1*	0
477 G	0.0036	405	0.0036	0	*0*	*1*	0
478 V	0.072	406	0.072	0.018	*0*	*1*	0
479 A	0.019	407	0.019	0.041	*0*	*1*	0
480 I	0.028	408	0.028	0.036	*0*	*1*	0
481 V	0.2	409	0.2	0.018	*0.001*	*1*	0
482 D	0.0024	410	0.0024	0	*0*	*1*	0
483 L	0.0035	411	0.0035	0	*0*	*1*	0
484 S	0.025	412	0.025	0.006	*0*	*1*	0
485 L	0.078	413	0.078	0.064	*0*	*1*	0
486 D	0.0024	414	0.0024	0	*0*	*1*	0
487 D	0.0023	415	0.0023	0	*0*	*1*	0
489 R	0.0029	417	0.0029	0	*0*	*1*	0
490 G	0.015	418	0.015	0.015	*0*	*1*	0
491 S	0.11	419	0.11	0.063	*0*	*1*	0
492 C	0.0035	420	0.0035	0	*0*	*1*	0
493 T	0.068	421	0.068	0.095	*0*	*1*	0
494 G	0.046	422	0.046	0.038	*0*	*1*	0
495 D	0.028	423	0.028	0.039	*0*	*1*	0
496 K	0.011	424	0.011	0.007	*0*	*1*	0
497 Y	0.028	425	0.028	0.002	*0*	*1*	0
498 P	0.0029	426	0.0029	0	*0*	*1*	0
499 I	0.033	427	0.033	0.063	*0*	*1*	0
500 L	0.0035	428	0.0035	0	*0*	*1*	0
501 R	0.013	429	0.013	0.009	*0*	*1*	0
502 A	0.0017	430	0.0017	0	*0*	*1*	0
503 A	0.034	431	0.034	0.069	*0*	*1*	0
504 K	0.021	432	0.021	0.038	*0*	*1*	0
505 Y	0.014	433	0.014	0.027	*0*	*0.999*	0
506 R	0.035	434	0.035	0.019	*0*	*1*	0
507 L	0.041	435	0.041	0.033	*0*	*1*	0

value for positions under purifying selection highlighted by purple
significant p value of FEL test in red
